# Extracellular vesicle microRNA and protein cargo profiling in three clinical-grade stem cell products reveals key functional pathways

**DOI:** 10.1016/j.omtn.2023.03.001

**Published:** 2023-03-09

**Authors:** Ramana Vaka, Sandrine Parent, Yousef Risha, Saad Khan, David Courtman, Duncan J. Stewart, Darryl R. Davis

**Affiliations:** 1 University of Ottawa Heart Institute, Division of Cardiology, Department of Medicine, University of Ottawa, Ottawa, ON K1Y4W7, Canada; 2 Department of Cellular and Molecular Medicine, University of Ottawa, Ottawa, ON K1H8M5, Canada; 3 Ottawa Hospital Research Institute, Division of Regenerative Medicine, Department of Medicine, University of Ottawa, Ottawa, ON K1H8L6, Canada

**Keywords:** MT: Special Issue - Exploiting Extracellular Vesicles as Therapeutic Agents, stem cells, extracellular vesicles, microRNA, proteins, functional enrichment

## Abstract

The cell origin-specific payloads within extracellular vesicles (EVs) mediate therapeutic bioactivity for a wide variety of stem cell types. In this study, we profiled the microRNA (miRNA) and protein cargos found within EVs produced by three clinical-grade stem cell products of different ontogenies being considered for clinical application, namely bone marrow-derived mesenchymal stromal cells (BM-MSCs), heart-derived cells (HDCs), and umbilical cord-derived MSCs (UC-MSCs). Although several miRNAs (757) and proteins (420) were found in common, each producer cell type expressed unique miRNA profiles when the most highly expressed transcripts were compared. Differential expression analysis revealed that BM-MSCs and HDCs were quite similar, while UC-MSCs had the greatest number of unique miRNAs and proteins. Despite these differences, all three EVs promoted cell adhesion/migration, immune response, platelet aggregation, protein translation/stabilization, and RNA processing. EVs from BM-MSCs were implicated in apoptosis, cell-cycle progression, collagen formation, heme pigment synthesis, and smooth muscle differentiation, while HDC and UC-MSC EVs were found to regulate complement activation, endopeptidase activity, and matrix metallopeptidases. Overall, miRNA and protein profiling reveal functional differences between three leading stem cell products. These findings provide a framework for mechanistic exploration of candidate therapeutic molecules driving the salutary effects of EVs.

## Graphical abstract

**Figure:**
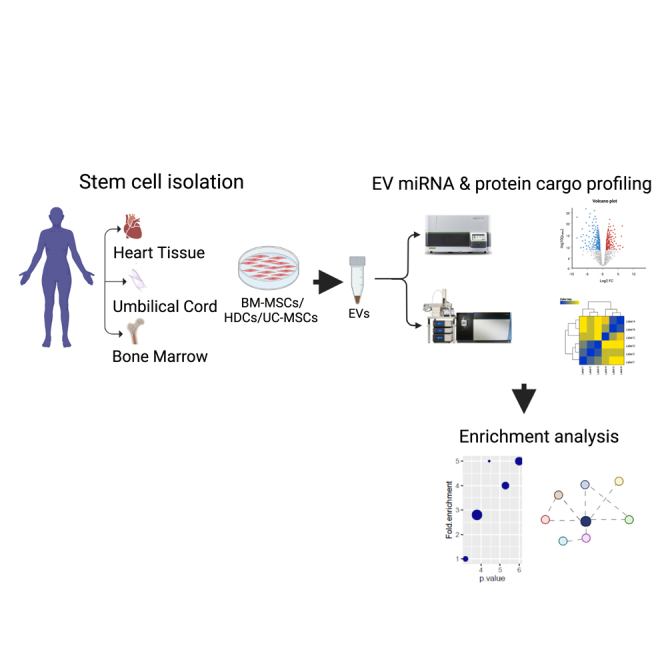

## Teaser

Comparison of three clinical-grade stem cell products (bone marrow-derived or umbilical cord mesenchymal stromal cells and heart-derived cells) highlights the differences in their extracellular vesicle microRNA and protein cargo, and the bioinformatic analysis on this cargo reveals important insights on the mechanistic understanding of these cell products.

## Introduction

Cell therapies are increasingly being investigated as novel treatments in disease models and patients. Thus far, clinicaltrials.gov reports 35,000+ clinical trials that assess cell therapies in various disease conditions. Recent work has shown these therapeutic benefits are driven by extracellular vesicles (EVs) secreted by transplanted cells.^1^^,^^2^^,^^3^^,^^4^^,^^5^ EVs are small vesicles secreted by nearly every cell in the body. Initially, vesicles were considered to be only a waste disposal system, but accumulating evidence has shown that EVs carry functional cargo (i.e., proteins, RNA, and lipids) that mediate cell-to-cell signaling.^6^ Since the initial publication of reports outlining the physiological significance of EVs,^7^^,^^8^^,^^9^^,^^10^^,^^11^^,^^12^ the number of studies characterizing cargo and functional relevance in preclinical disease models has increased.

The payloads within EVs are thought to be dependent upon tissue cell source as transcripts and proteins differ between studies. But how much of that variability can be attributed to biology as opposed to methodological issues (such as different media/EV collection conditions or protein/transcript quantification + analysis) is not clear. To date, there have been very few direct comparisons between EVs of different origins that use the same methodology.^13^^,^^14^ Despite these apparent differences, EV treatment often has similar salutary effects that are usually attributed to different anti-fibrotic or anti-inflammatory moieties. If true, these cargo differences represent an opportunity to engineer more potent EV pharmaceuticals that can tackle the myriad of known and unknown pathways underlying different diseases or, at the very least, build in additional redundancy to target critical drivers of pathology.

To address this challenge, we profiled the cargo within EVs isolated from three different producer cell lines being considered for clinical applications, namely bone marrow-derived mesenchymal stromal cells (BM-MSCs), heart-derived cells (HDCs), and umbilical cord-derived MSCs (UC-MSCs). To enhance reproducibility and insight into eventual clinical translation, all cell lines were produced in a clinical-grade cell manufacturing facility to good manufacturing practice (GMP) standards using sourced materials. For over 20 years, BM-MSCs have been studied, with multiple reports supporting utility in several diseases.^15^^,^^16^^,^^17^^,^^18^^,^^19^^,^^20^^,^^21^^,^^22^ The BM-MSC lines used in this study were sourced from qualified normal donors, thus avoiding potential adverse effects of medical comorbidities on therapeutic efficacy. Compared with BM-MSCs, UC-MSCs display a more consistent phenotype, higher yields, more rapid growth, and potentially higher therapeutic efficacy (potency).^23^^,^^24^^,^^25^ Although there are no head-to-head studies demonstrating therapeutic efficacy, the origin and physiological function of UC-MSCs likely lead to very different EV cargo. HDCs represent an emerging CD45−/CD105+ cell type culture directly from small pieces of heart tissue that confer protection in heart failure^26^^,^^27^^,^^28^ and possibly systemic diseases.^29^ Forensic analysis has shown that HDCs have no detectable contribution from extra-cardiac sources,^30^ thus providing the opportunity to identify unique transcripts and proteins that might be more cardiac specific.

Therefore, we profiled the microRNA (miRNA) and protein cargo within EVs collected from three clinical-grade cell lines using multiplex fluorescent oligonucleotide-based miRNA detection and liquid chromatography-mass spectrometry (LC-MS), respectively. Functional and pathway enrichment analysis was used to probe for important and unique differences attributable to EV cell source.

## Results

### Cell culture and EV characterization

**Figure 1:**
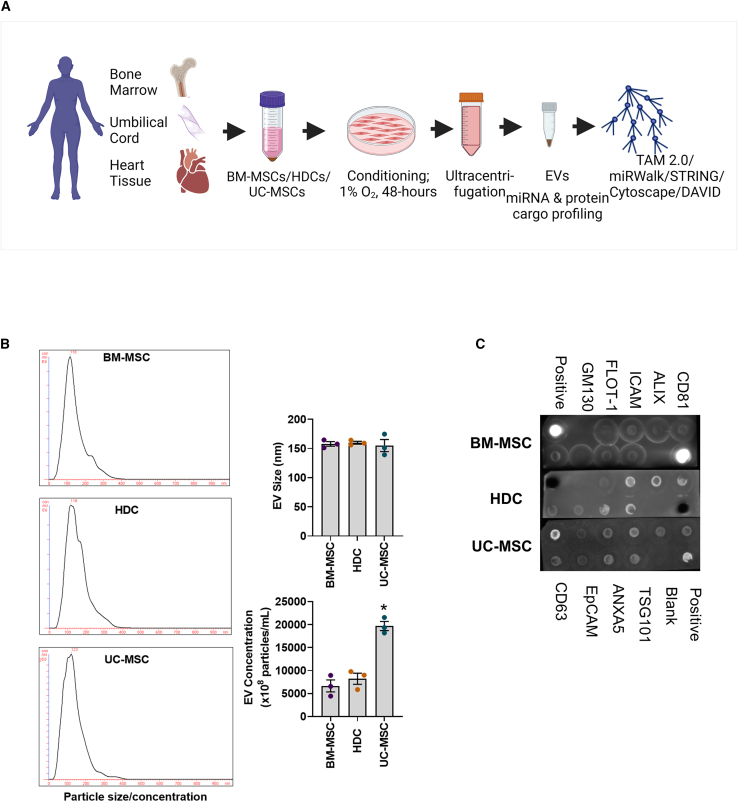
EV characterization (A) Schematic of experimental methodology utilized in the study. (B) Representative plots of nanoparticle tracking analysis of EVs. Nanoparticle tracking analysis showed that the concentration and size of EVs isolated from all 3 cell types were representative of accepted definitions for EV identity. While the EV concentration was similar in BM-MSCs and HDCs, UC-MSCs produced 2-fold higher EVs in comparison. (C) Proteomic antibody array showed the presence of 8 known EV markers (ICAM, ALIX, CD81, CD63, EPCAM, ANXAS, TSG101, FLOT-1) and the absence of bands for *cis*-Golgi marker (GM130) suggesting that EV preparations were free of cellular contaminants. All data are presented as individual and mean values ± SEM, n = 3 biological replicates; each filled circle on the bar graph represents one data point from one unique biological replicate. Differences between cell types were analyzed by one-way ANOVA. ∗p < 0.05 compared with BM-MSC or HDC EVs. BM-MSC, bone marrow-derived mesenchymal stromal cells; HDC, heart-derived cells; UC-MSC, umbilical cord-derived mesenchymal stromal cells.

A schematic of the experimental methodology is shown in Figure 1A. All three cell products were manufactured to clinical release standards and have been previously characterized for their surface marker identity^31^^,^^32^ and tri-lineage differentiation.^33^^,^^34^ Cell characterization was not repeated in the current study. The quality control parameters of cell products are shown in Table S1. EVs were isolated from conditioned media collected from cultured cells using differential ultracentrifugation. The sizes of EVs isolated from all 3 cell lines were representative of accepted definitions for EV identity^35^ with no differences attributable to producer cell line (Figure 1B). While BM-MSCs and HDCs produced similar amounts of EVs, UC-MSCs yielded 2-fold more EVs (p < 0.05 vs. BM-MSCs or HDCs). All 3 EV preparations expressed common EV surface markers (ALIX, ANXAS, CD81, CD63, EPCAM, FLOT-1, TSG101, ICAM) without evidence for cellular contamination (GM130; Figure 1C).

### EV miRNA and protein cargo profiling within BM, heart, and UC EVs

**Figure 2:**
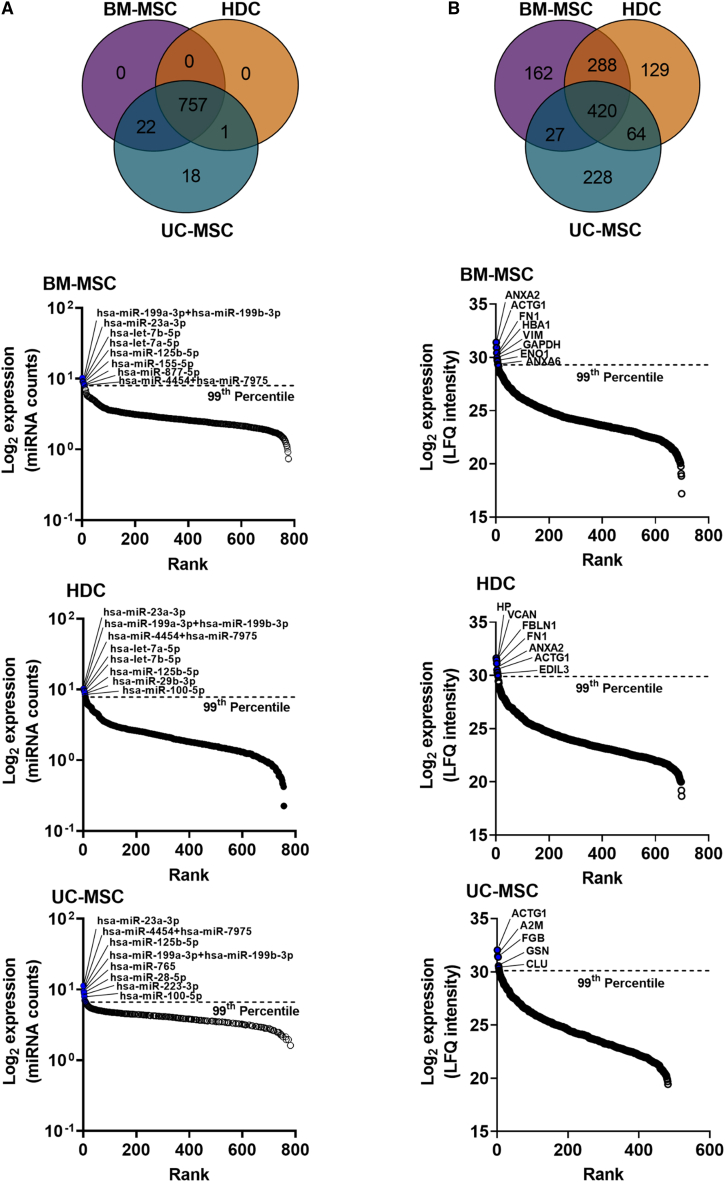
MicroRNA and proteomic profiling of EVs NanoString miRNA and LC-MS protein LFQ data were processed for background subtraction, normalization, and log2 transformation using ROSALIND and Perseus (https://maxquant.net/perseus/), respectively. The normalized mean data were visualized using Venn diagrams and rank order plots. The highly abundant (above the 99^th^ percentile) miRNAs and proteins are highlighted (blue filled circles) on the rank order plots. (A) Venn diagram and rank order plots showing distinct number of miRNAs identified in three stem cell types. (B) Venn diagram and rank order plots showing distinct number of proteins identified in three stem cell types. The distinct highly abundant (above the 99^th^ percentile) miRNAs and proteins between cell types are visualized using in Venn diagrams shown in Figure S1. Data are presented as mean values, n = 3 biological replicates; each circle on the rank order plots represents one distinct miRNA or protein. miRNA, microRNA; LFQ, label-free quantitation; BM-MSC, bone marrow-derived mesenchymal stromal cells; HDC, heart-derived cells; UC-MSC, umbilical cord-derived mesenchymal stromal cells.

As shown in Figure 2A, 757 miRNAs were commonly expressed by all 3 EVs. Interestingly, UC-MSCs were found to express the most distinct EVs with 18 unique miRNAs. Within the highly expressed miRNAs (i.e., expression above the 99^th^ percentile), 4 miRNAs were found to be commonly expressed among all 3 EVs (miR-199a+miR-199b, miR-23a, miR-4454+miR-7975, miR-125b-5p). Two miRNAs (let-7a, let-7b) were shared between BM-MSC and HDC EVs, while 1 miRNA (miR-100) was found in both HDC and UC-MSC EVs (Figure S1A).

As shown in Figure 2B, 420 proteins were expressed in all 3 cell types. In contrast to the few miRNAs uniquely expressed, all 3 EVs were found to contain several unique proteins (i.e., 100+), with the greatest number of shared proteins found in BM-MSCs and HDCs. UC-MSCs expressed the greatest number of unique proteins. When ranked as highly expressed proteins, only ACTG1 protein was shared above the above 99^th^ percentile in EVs from 3 cell types, while ANXA2 and FN1 were shared between BM-MSC and HDC EVs (Figure S1B).

### Differential expression of miRNAs or proteins within BM, heart, and UC EVs

**Figure 3:**
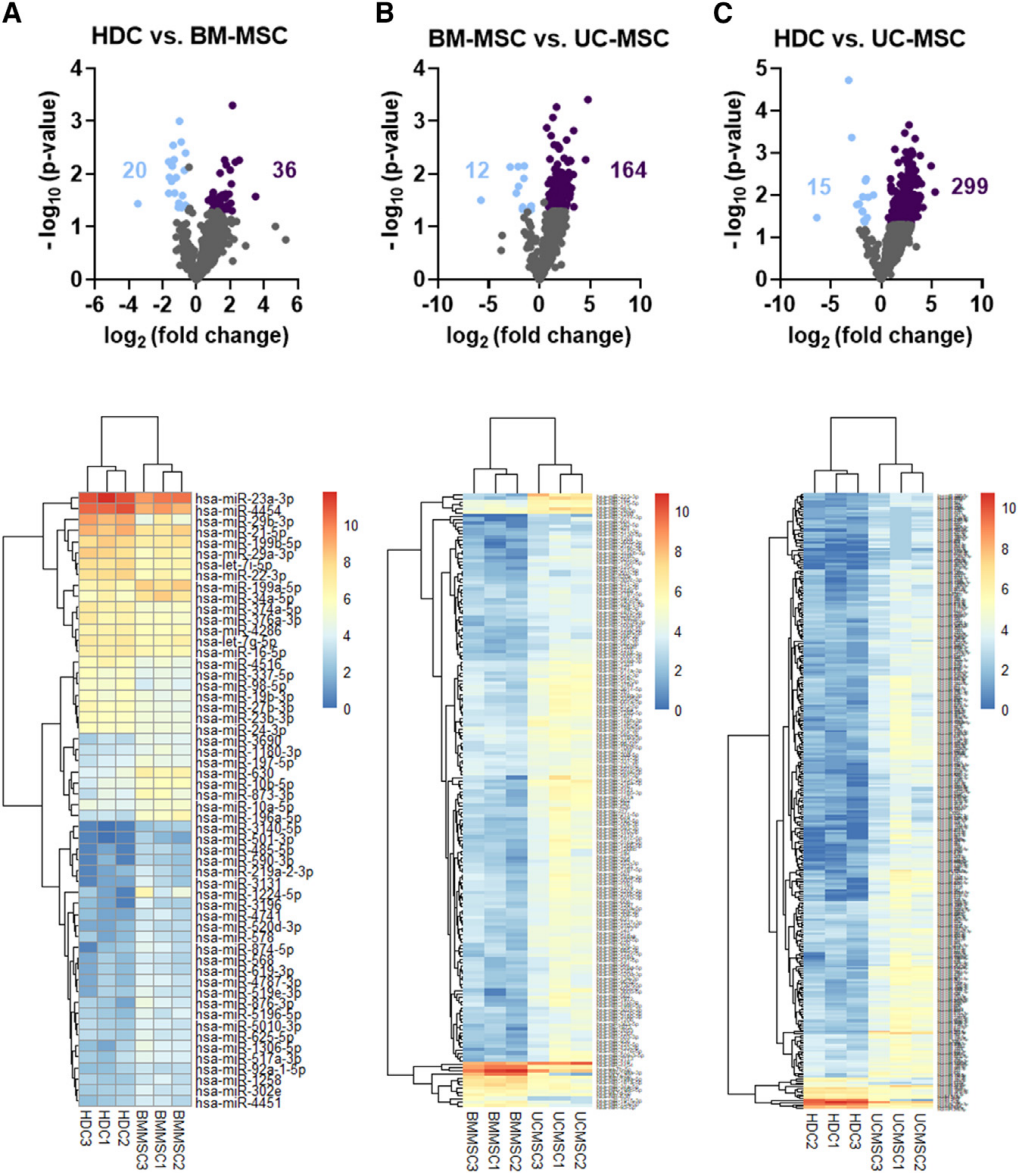
Differential expression of microRNAs from EVs Differential expression analysis of miRNAs between cell types was performed using ROSALIND t test method. p value adjustment was performed using the Benjamini-Hochberg method of estimating false discovery rates (FDRs). miRNAs were considered differentially expressed with a log2 fold change ≥ or ≤1.5 and p <0.05. Log2 fold change and p values were exported from ROSALIND to construct volcano plots and heatmaps using GraphPad Prism v.9.1 and RStudio (pheatmap package), respectively. (A) Volcano plot showing 20 downregulated and 36 upregulated miRNA transcripts, and heatmap showing the significant differentially expressed miRNAs in HDC vs. BM-MSC EVs. (B) Volcano plot showing 12 downregulated and 164 upregulated miRNA transcripts, and heatmap showing the significant differentially expressed miRNAs in BM-MSC vs. UC-MSC EVs. (C) Volcano plot showing 15 downregulated and 299 upregulated miRNA transcripts, and heatmap showing the significant differentially expressed miRNAs in HDC vs. UC-MSC EVs. The list of differentially expressed miRNAs is provided in Tables S1, S2, and S3. n = 3 biological replicates. BM-MSC, bone marrow-derived mesenchymal stromal cells; HDC, heart-derived cells; UC-MSC, umbilical cord-derived mesenchymal stromal cells.

**Figure 4:**
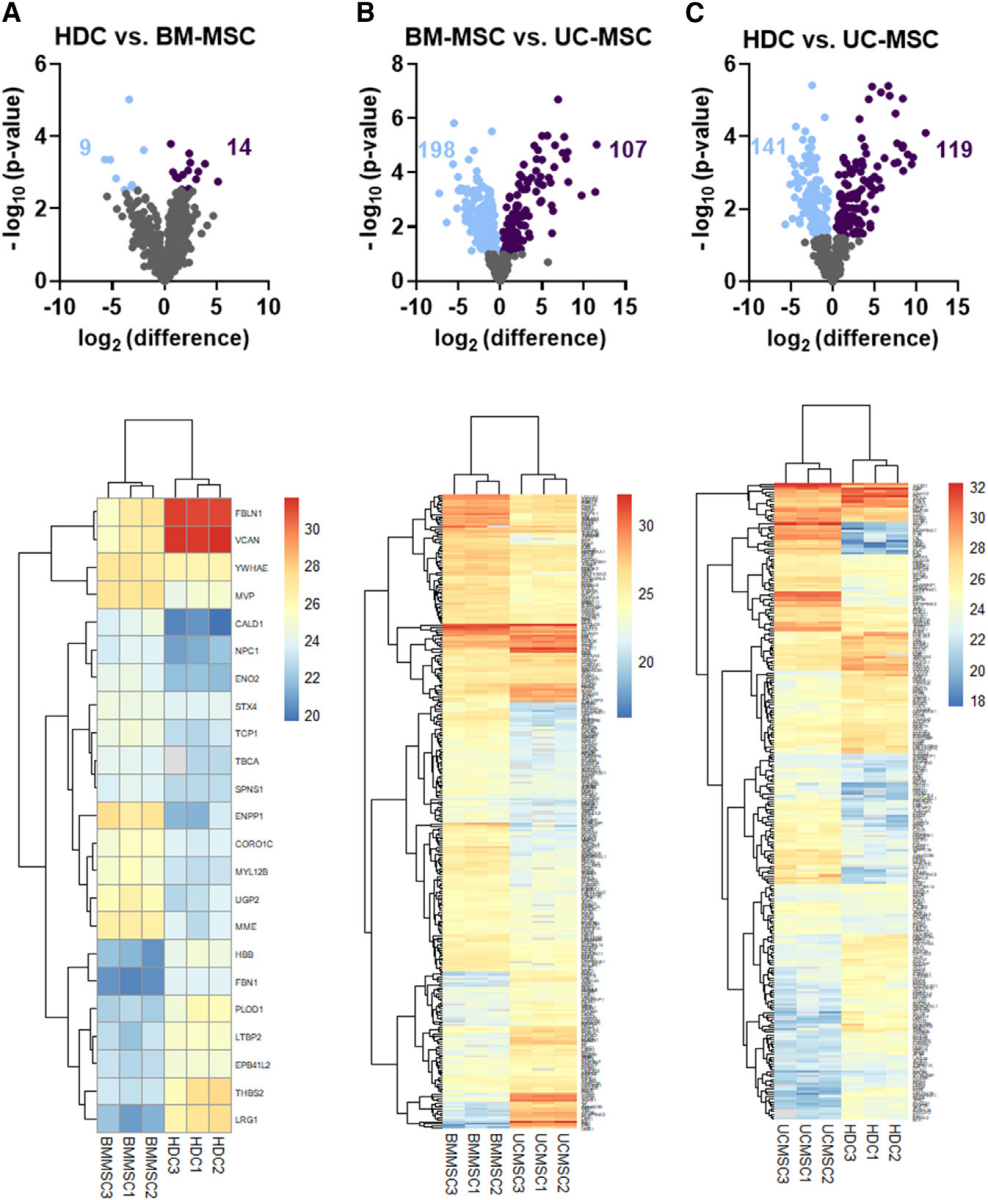
Differential expression of proteins from EVs Differential expression analysis of proteins between cell types was performed using Perseus (https://maxquant.net/perseus/). Proteins identified in at least 2 of 3 replicates were considered for analysis. Two-sample two-tailed Student’s t test with permutation-based FDR (FDR = 0.05, no. of randomizations = 250) was used to calculate statistical significance between cell types. The proteins were considered differentially expressed with a p <0.05. Log2 difference and p values were exported from Perseus to construct volcano plots and heatmaps using GraphPad Prism v.9.1 and RStudio (pheatmap package), respectively. (A) Volcano plot showing 9 downregulated and 14 upregulated proteins, and heatmap showing the significant differentially expressed proteins in HDC vs. BM-MSC EVs. (B) Volcano plot showing 198 downregulated and 107 upregulated proteins, and heatmap showing the significant differentially expressed proteins in BM-MSC vs. UC-MSC EVs. (C) Volcano plot showing 141 downregulated and 119 upregulated proteins, and heatmap showing the significant differentially expressed proteins in HDC vs. UC-MSC EVs. The list of differentially expressed proteins is provided in Tables S4, S5, and S6. n = 3 biological replicates. BM-MSC, bone marrow-derived mesenchymal stromal cells; HDC, heart-derived cells; UC-MSC, umbilical cord-derived mesenchymal stromal cells.

To probe the miRNA stoichiometry for differences in expression patterns, we compared differential miRNA expression using a 1.5-fold log2 threshold (Figure 3; Tables S2, S3, and S4). Expression of miRNA in BM-MSC and HDC EVs was largely similar with only 56 differentially regulated transcripts. In contrast, UC-MSC EV expression was markedly different with 176 and 314 differentially expressed miRNAs compared with BM-MSC and HDC EVs, respectively. Analysis of protein expression showed a similar pattern, with only 23 proteins showing differential expression when BM-MSC EVs were compared with HDC EVs (Figure 4; Tables S5, S6, and S7). UC-MSC EVs had 305 and 260 differential expressed proteins when compared with BM-MSC and HDC EVs, respectively.

### Functional analysis of miRNA expression within BM, heart, and UC EVs

**Figure 5:**
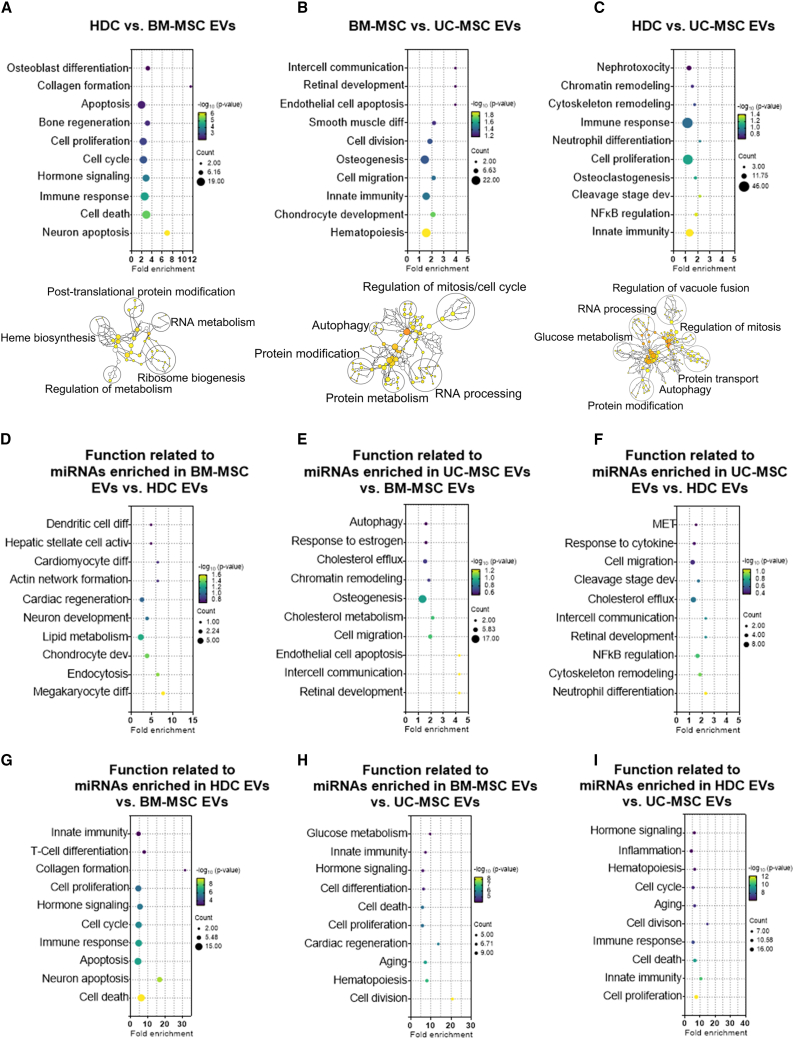
Functional enrichment analysis of EV miRNA cargo TAM 2.0 (http://www.lirmed.com/tam2/) was used to perform miRNA functional enrichment analysis. The list of mature miRNA names from normalized ROSALIND data of all differentially expressed miRNAs and up- or downregulated miRNAs was used as input (overrepresentation, p ≤ 0.05) for enrichment analysis. TAM 2.0 data output: enriched term, fold enrichment, p values, and miRNA count are graphed as bubble plots using GraphPad Prism v.9.1. Further, Gene Ontology (biological process) enrichment analysis on experimentally validated mRNA targets of differentially expressed miRNAs was performed. The differentially expressed miRNA list was used as an input in the miRWalk v.3 (http://mirwalk.umm.uni-heidelberg.de/) (interaction probability score = 0.95, miRTarBase, 3′ UTR) to obtain the target information (Tables S8–S10) and then imported to Cytoscape to visualize networks (Figure S2), and enrichment analysis was performed using Cytoscape plugin BINGO (overrepresentation, hypergeometric test with Benjamini-Hochberg FDR correction, p = 0.05). (A–C) The top 10 significantly enriched biological functions and Gene Ontology biological processes of all differentially expressed miRNAs among three cell products. (D–I) The top 10 significantly enriched biological functions of enriched miRNAs in one cell type compared with other cell types. Significantly enriched terms related to transcription factors and tissue specificity are presented in Figure S3. The list of differentially expressed miRNA-mRNA targets is provided in the Tables S7–S9. n = 3 biological replicates. Activ, activity; BM-MSC, bone marrow-derived mesenchymal stromal cells; Dev, development; Diff, differentiation; HDC, heart-derived cells; MET, mesenchymal-epithelial transition; UC-MSC, umbilical cord-derived mesenchymal stromal cells.

As shown in Figure 5, HDC EVs expressed miRNA transcripts implicated in apoptosis, collagen formation, osteoblast differentiation, and cell proliferation when compared with EVs from BM-MSCs. Gene Ontology (GO) enrichment analysis demonstrated that miRNA-mRNA targets were significantly enriched in pigment biosynthesis, mRNA catabolism, and RNA processing. miRNAs highly expressed in HDC EVs were associated with differentiation, cardiac regeneration, and differentiation, while miRNAs highly expressed in BM-MSC EVs were involved in collagen formation, cellular proliferation, and cell death.

EVs from BM-MSCs were enriched in transcripts implicated in apoptosis, hematopoiesis, and retinal development when compared with EVs from UC-MSCs. GO analysis of transcripts from BM-MSC EVs suggest involvement in glucose metabolism, protein transport, RNA processing, and vacuole transport. miRNAs upregulated in BM-MSC EVs were involved in apoptosis, cell migration, and osteogenesis, while miRNAs enriched within UC-MSCs were involved in aging, cardiac regeneration, and proliferation.

When HDC EVs were compared with UC-MSCs EVs, miRNAs were implicated in development, immune regulation, and proliferation. GO analysis of transcripts from HDC EVs were implicated in glucose metabolism, protein transport, RNA processing, and vacuole transport. Highly expressed miRNAs in HDC EVs were involved in mesenchymal-to-epithelial transition, regulation of nuclear factor κB (NF-κB), and development, while highly expressed miRNAs in UC-MSCs were involved in aging, inflammation, and proliferation. The downstream transcription factors and tissue associated with these functions for EVs from all 3 producer cell lines are shown in Figure S3.

### Functional analysis of protein expression in BM, heart, and UC EVs

**Figure 6:**
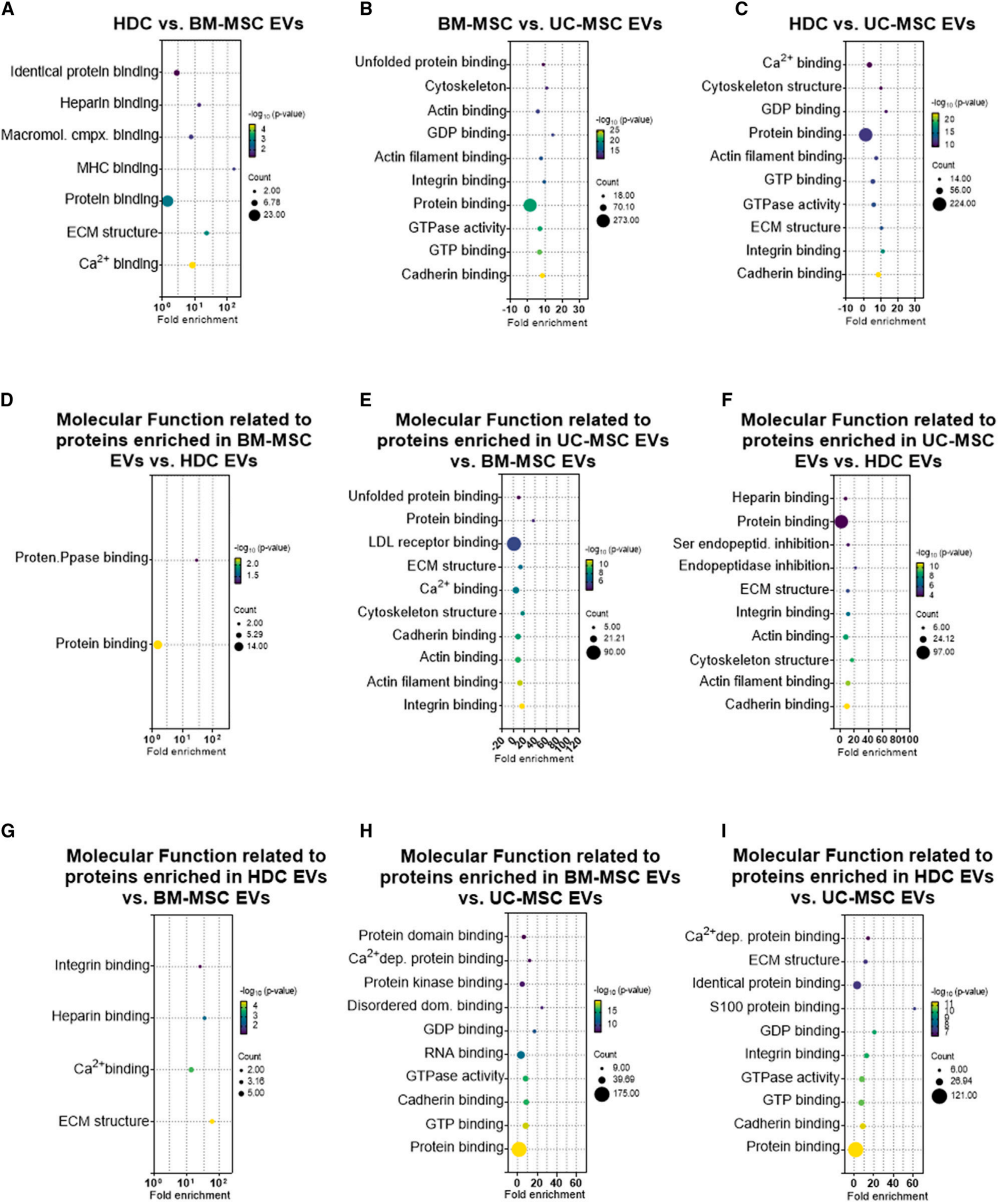
Functional enrichment analysis of EV protein cargo Functional enrichment analysis of the whole proteome and differentially expressed proteins was performed using DAVID v.6.8 (https://david.ncifcrf.gov/) (*Homo sapiens* proteome as a background, Fisher’s exact test with multiple testing by the Benjamini-Hochberg method with adjusted p = 0.05). The significantly enriched functional Gene Ontology (GOP) terms of biological process (BP), cellular component (CC), and molecular function (MF) and Kyoto Encyclopedia of Genes and Genomes (KEGG) pathways were extracted and graphed as bubble plots using GraphPad Prism v.9.1. (A–C) The top 10 significantly enriched molecular functions of all differentially expressed proteins among three cell products. (D–I) The top 10 significantly enriched molecular functions of enriched proteins in one cell type compared with other cell types. The top 10 significantly enriched biological processes, cellular functions, and KEGG pathways in differentially expressed proteins and in enriched proteins in one cell type compared with other cell types are presented in Figures S4 and S5. n = 3 biological replicates. BM-MSCs, bone marrow-derived mesenchymal stromal cells; ECM, extracellular matrix; GDP, guanosine diphosphate; GTP, guanosine triphosphate; HDC, heart-derived cells; LDL, low-density lipoprotein; Macromol.cmpx, macromolecule complex; MHC, myosin heavy chain; Protein. Ppase, protein phosphatase; UC-MSC, umbilical cord-derived mesenchymal stromal cells.

Enrichment analysis of the protein cargo within EVs revealed several terms related to biological processes, cellular components, and molecular functions for each cell source (Table S11). The top 10 biological processes of whole proteome for each source are shown in Table S12. The top 10 biological processes, cellular components, and Kyoto Encyclopedia of Genes and Genomes (KEGG) pathways of differential expressed proteins are shown in Figures S4 and S5. Unsurprisingly, many of the top terms were shared and related to EV biology or cellular binding (i.e., cell adhesion, extracellular exosome, focal adhesion, cadherin binding, and protein binding). When EVs from different cell types were compared, each producer cell line imparted several unique terms, with the greatest degree of homology shared between BM-MSC and HDC EVs. When HDC EVs were compared with BM-MSC EVs, functional pathways were largely involved in calcium ion binding, cell binding, and extracellular matrix assembly (Figure 6). Unsurprisingly, proteins overexpressed in EVs produced by BM-MSCs were more apt to be implicated in heparin or integrin binding. When BM-MSC EVs were compared with UC-MSC EVs, differentially expressed proteins were involved in actin organization, cadherin binding, and cellular adhesion. Proteins highly expressed within BM-MSC EVs were involved in protein + RNA binding, while UC-MSC EVs’ highly expressed proteins were in extracellular matrix binding/structure and low-density lipoprotein (LDL) receptor biology. When HDC EVs were compared with UC-MSC EVs, proteins were implicated in calcium and protein binding.

### Protein-protein interactions imparted by BM, heart, and UC EVs

Proteins regulate biological processes through functional or physical interactions with other proteins. Using protein-protein interaction network analysis, we probed for potential interactions between differentially expressed proteins. As shown in Figure S6, this analysis revealed several nodes and interaction pairs, with the highest degree of homology shared between HDC and BM-MSC EVs. Key nodes were then used to extract hub genes for cluster analysis which yielded 1 cluster from the HDC vs. BM-MSC EV network, 9 clusters from the BM-MSC vs. UC-MSC EV network, and 7 clusters from the HDC vs. UC-MSC EV network.

Using a topological scoring system,^36^ we found that the HDC vs. BM-MSC EV network did not contain significant clusters, indicating a high degree of homology.

**Figure 7:**
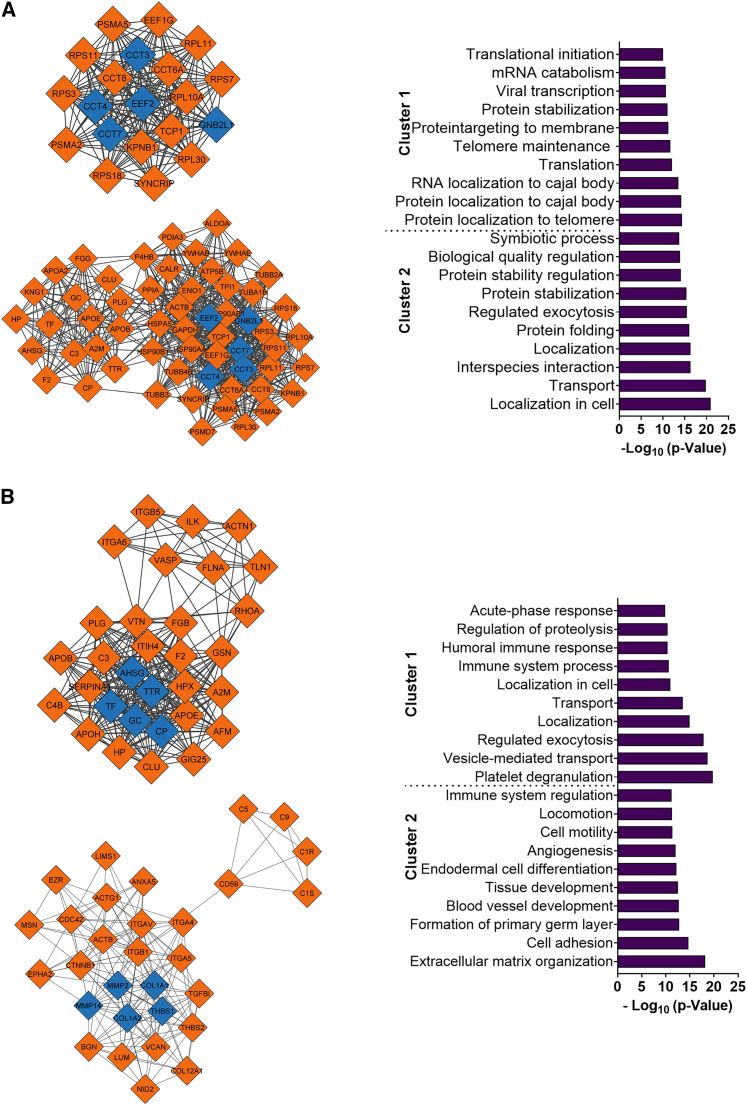
Protein-protein interaction (PPI) network and GO-BP enrichment analysis of differentially expressed proteins in EVs STRING v.11.5 (https://string-db.org/cgi/input.pl) was used to analyze protein-protein interaction (PPI) analysis of differentially expressed proteins (medium confidence score = 0.4). STRING PPI network was used for cluster and GO (BP) enrichment analysis using Cytoscape plugin MCODE (https://apps.cytoscape.org/apps/MCODE) (degree cutoff = 2, cluster finding: haircut, node score cutoff = 0.2, K-core = 2, maximum [max.] depth = 100). CytoHubb plugin was used to extract to hub genes in the PPI network clusters. (A) Significant clusters from the PPI network and enriched GO-BP terms of BM-MSC vs. UC-MSC differentially expressed proteins. (B) Significant clusters from the PPI network and enriched GO-BP terms of HDC vs. UC-MSC differentially expressed proteins. The orange diamonds indicate nodes, gray lines indicate edges, and the blue diamonds indicate hub node genes. The PPI networks of differentially expressed proteins among cell types are presented in Figure S6.

Two significant clusters were found within the comparison between BM-MSC and UC-MSC EVs (topological score = 19.36, 20 nodes, 184 interaction pairs). Analysis of cluster 1 revealed hub genes related to chaperon-containing proteins (CCT3, degree = 32; CCT7, degree = 34; CCT8, degree = 26) and ribosomal protein subunits (RPS3, degree = 28; RPL11, degree = 25; Figure 7A). String enrichment analysis indicated that the top biological processes were related to protein localization to telomeres (GO: 1904851), protein localization to Cajal bodies (GO: 1904871), and telomerase localization to Cajal bodies (GO: 1904874). Cluster 2 contained 57 nodes and 668 interaction pairs, with the top 5 hub genes being related to chaperon containing proteins (CCT3, degree = 32; CCT4, degree = 33; CCT7, degree = 34), nucleotide-binding protein (GNB2L1, degree = 34), and translation elongation factor (EEFF2, degree = 39). The top 2 biological process terms identified related to cell localization (GO: 0051649) and interspecies interaction between organisms (GO: 0006810).

The comparison between HDC and UC-MSC EVs yielded 2 clusters, with cluster 1 (score = 17.6) containing 31 nodes and 264 interaction pairs (Figure 7B). The top 5 hub genes within cluster 1 were related to copper binding (CP, degree = 22), iron biding transport (TF, degree = 22), thyroid hormone-binding (TTR, degree = 22), and vitamin D transport (GC, degree = 22). The top biological process terms within cluster 1 were implicated in exocytosis (GO: 0045055), platelet degranulation (GO: 0002576), and vesicle transport (GO: 0016192). Cluster 2 contained 30 nodes and 169 interaction pairs. The top hub genes within this cluster were related to collagen (COL1A1, degree = 8; COL1A2, degree = 17), glycoprotein (THBS1, degree = 17), and matrix metallopeptidases (MMP2, degree = 19; MMP14, degree = 16). String enrichment analysis within this cluster showed that proteins mediated cell adhesion (GO: 0007155), extracellular matrix organization (GO:0030198), and formation of the primary germ layer (GO: 0001704).

## Discussion

Recent work suggests that the therapeutic effects of cell treatment are partially attributable to delivery of the biological cargo within EVs secreted by transplanted cells.^37^ In this study, we analyzed the miRNA transcriptome and proteome from three leading stem cell products under clinical investigation for several diseases, including cardiovascular diseases and inflammation-mediated pathologies.

Interestingly, we found that the cargo within EVs was often much more similar than different. Several miRNAs and proteins commonly expressed in all three cell types were related to cell adhesion, cell proliferation, immune response, platelet aggregation, and protein translation/stabilization. Although many of the same miRNAs and proteins were present in EVs from all three cell types, stoichiometry between different EVs was often markedly different. For example, miR-29b is found to be highly expressed in HDC EVs (ranked #7) compared with other cell types: BM-derived stromal cell EVs (ranked #51) or UC-derived stromal cell EVs (ranked #42). Despite these differences, EVs from all three cell types highly expressed six miRNAs (miR-23a, -125b, -199a, -199b, -4454, and -7975). Functional enrichment analysis of these six miRNAs (data not shown) reveals that they are involved in endocytosis, immune response, inflammation, osteogenesis, osteoblast differentiation, and cell proliferation. Previous studies have shown that stromal cells of mesenchymal origin and their EVs regulate inflammation and bone formation and angiogenesis.^38^^,^^39^^,^^40^^,^^41^ These salutary effects can partially be attributed to abundant expression of these 6 common miRNAs in these cells. All three EVs were found to contain high levels of the ubiquitous protein actin gamma 1 (*ACTG1*), which is involved in cytoskeleton organization. When the differential expression of miRNAs and proteins was analyzed, BM-MSC and HDC EVs appeared to be more similar than UC-MSC EVs, a finding that is consistent with their similar physiologic purpose (i.e., organ repair vs. reproductive biology).

There were also transcripts and proteins characteristic of each EV type. HDC EVs were the only source for miR-29b, EGF-like repeats and discoidin domains 3 (*EDIL3*), Fibulin 1 (*FBLN1*), haptoglobin (*HP*), and Versican (*VCAN*). BM-MSC EVs were marked by miR-155, miR-877, annexin A6 (*ANXA6*), enolase 1 (*ENO1*), glyceraldehyde-3-phosphate dehydrogenase (*GAPDH*), hemoglobin subunit alpha 1 (*HBA1*), and vimentin (*VIM*). UC-MSC EVs were the only source for miR-765, miR-28, miR-223, α-2-macroglobulin (*A2M*), clusterin (*CLU*), fibrinogen β chain (*FGB*), and gelsolin (*GSN*).

When compared with the few transcriptional profiling reports in the literature,^42^^,^^43^^,^^44^^,^^45^ our findings mirrored those studies. For example, a recent miRNA profiling study of UC-MSC EVs found several highly abundant commonalities (miRs-16, -21, -23b, -25, -34, -146a, and -222) that predicted involvement in immune regulation and proliferation.^42^ Baglio et al. showed that BM-MSC EVs contained several miRNA species (miRs-21, -22, −26a, -10b, -99b, -125b, and -148a)^46^ that were found in the top miRNAs expressed in BM-MSC EVs in our study. Interestingly, a recent study by Liao et al. highlighted the functional role of BM-MSC EV-derived miR-122 in promoting osteoblast proliferation.^47^ We found miR-122 within the top miRNAs in our BM-MSC EVs, and functional enrichment analysis confirmed overrepresentation of osteoblast and osteogenesis terms.

In terms of proteomic comparisons, Wang et al. recently performed a comprehensive proteomic analysis of EVs from adipose, BM, and UC-MSCs. In this report, BM-MSC EVs expressed 771 proteins that were largely associated with cadherin binding and protein biosynthesis, while UC EVs expressed 431 proteins that were associated with calcium ion binding, extracellular matrix formation, and integrin signaling/adhesion.^48^ Sixty proteins were found in both EV sources. While their functional enrichment findings are largely in line with our results, one distinction is that our analysis revealed slight variations in protein abundance that are likely attributable to differences in donor selection, cell manufacturing, and EV isolation.

It is challenging to speculate which producer cell line might offer superior therapeutic efficacy given the complexity of several (magnitude of 100s) biologically active molecules found within EVs. The functional comparison between different subtypes suggests that some EVs may be better suited toward specific applications (e.g., anti-fibrotic effects by HDC EVs in reducing post-infarct scarring). Understanding the differential expression of proteins or transcripts opens the possibility for engineering producer cell lines to boost functional effects (e.g., increasing miR-146a in BM-MSCs to improve BM-MSC EV pro-angiogenic effects). Akin to using a mixture of different grass seeds to reseed a lawn, combining different EV subtypes as a single therapy to boost efficacy might be the most practical and cost-effective way to improve treatment outcomes. This would ensure that different signaling pathways are recruited to maximize benefit. From our analysis, the combination of UC-MSC EVs with either BM-MSC EVs or HDC EVs would be the most logical choice given the miRNA and protein similarities between BM-MSC and HDC EVs.

Our study has several limitations that include (1) a small sample size, limited to three biological replicates from each cell product; (2) reliance on candidate miRNA profiling that includes only commonly expressed miRNAs; (3) target network and enrichment analysis confined to experimentally validated 3′ UTR targets, although recent work has shown that targeting may expand to the 5′ UTR and coding sequence regions of mRNA; (4) no evidence for other non-coding RNAs, which may play important roles in mediating EV effects; (5) no functional tests to confirm if the observed differences in protein or transcripts translate into meaningful differences in function; and (6) inclusion of 1% platelet lysate during generation of conditioned media by UC-MSCs, which may have led to trace contamination of platelet-derived vesicles. Future work will be needed to expand this work to other non-coding RNAs, to other targeting regions, and to confirmatory validation in functional tests using up- or downregulation of candidate targets.

In summary, we profiled miRNA and protein cargo from three leading EV products under investigation. We identified several common miRNA transcripts and proteins and some unique that were in each donor cell type. Further, we found that these unique cargos display distinct functional enriched categories and pathways that offered mechanistic understanding of existing studies showing EV-mediated benefit. Overall, our study characterized EV miRNA and proteome and displayed functional significance of this cargo, which will further a great mechanistic understanding of the EV therapeutic effects in various models of disease.

## Materials and methods

### Cell culture and EV isolation

BM-MSCs were isolated from BM samples collected from healthy volunteers enrolled in the Cellular Immunotherapy for Septic Shock (CISS) trial under protocols approved by the Ottawa Hospital Research Ethics Board.^33^ HDCs were isolated from atrial appendage tissue collected from patients undergoing clinically indicated surgery under protocols approved by the University of Ottawa Heart Institute Research Ethics Board.^32^^,^^49^^,^^50^ A protocol was developed to isolate and culture UC-MSCs from UCs collected during scheduled C-sections performed at The Ottawa Hospital under protocols approved by the Ottawa Hospital Research Ethics Board.^33^ All cell products were manufactured to clinical-grade release standards in Biospherix units at The Ottawa Hospital Cell Manufacturing Facility. BM-MSCs were cultured in NutriStem XF media (Sartorius) under 21% oxygen conditions.^33^ HDCs were cultured in NutriStem XF media at 5% oxygen conditions.^32^ UC-MSCs were cultured in Dulbecco’s modified Eagle medium (Thermo Fisher Scientific) with 10% clinical grade platelet lysate (Mill Creek Life Sciences) at 5% oxygen conditions. When cells reached 70% confluency, culture media were replaced with condition media (BM-MSCs and HDCs: NutriStem XF basal media; UC-MSCs: Dulbecco’s modified Eagle medium with high glucose and 1% platelet lysate). After 48 h of conditioning at 1% oxygen, media were collected for EV isolation. EVs were isolated using ultracentrifugation (10,000*g* × 30 min and 100,000*g* × 3 h).^51^^,^^52^

### Nanoparticle tracking system

The size and concentration of EV preparations were analyzed using NanoSight LM10 equipped with a blue laser (488 nm, 70 mW) with an sCMOS camera. Briefly, 1 μL final pellet suspension was diluted at 1:1,000 in saline, and 500 μL was loaded into the sample chamber. Three videos of 60 s were recorded for each sample. Data analysis was performed with NTA 3.0 software (Nanosight).

### EV proteomic antibody array

EV markers were characterized by using proteomic array as per the manufacturer’s recommendations (EXORAY200A; System Biosciences). In brief, 50 μg EV lysate was incubated with labeling reagent for 30 min, followed by incubation with membrane precoated antibodies for 8 known EV markers. After overnight incubation, detection buffer added before membranes were washed and scanned with X-ray imager.

### miRNA expression assay

miRNA was isolated from EVs using the appropriate miRNA isolation kit (Qiagen). 100 ng miRNA was used for the nCounter miRNA sample preparation reactions. Sample preparation was performed according to the manufacturer’s instructions. All hybridization reactions were incubated at 65°C for a minimum of 18 h. Hybridized probes were purified and counted on the nCounter Prep Station and Digital Analyzer. For each assay, a high-density scan (600 fields of view) was performed. The miRNA count data obtained from NanoString were analyzed by ROSALIND (https://rosalind.bio/). Background subtraction was performed based on POS_A probe correction factors, and normalization was performed using the geometric mean of each code set from the positive control normalization and code set normalization.

After normalization, fold changes were calculated, and comparisons between two EV types were assessed using the ROSALIND t test method. p value adjustment was performed using the Benjamini-Hochberg method of estimating false discovery rates (FDRs). Log2 fold changes, p values, and normalized log2 count data were exported from ROSALIND to construct volcano plots and heatmaps using GraphPad Prism v.9.1 and RStudio (pheatmap package), respectively.

miRNA functional enrichment analysis was analyzed using TAM 2.0 (http://www.lirmed.com).^53^^,^^54^ The list of mature miRNA names from the normalized ROSALIND data was used as input (overrepresentation, p ≤ 0.05). Three functional enrichment categories were extracted (i.e., function, tissue specificity, and transcription factor) to ascribe the functional significance of the miRNA cargo found within EVs. To delineate functional differences pertaining to miRNA cargo across all 3 EV types, we performed enrichment analysis using the list of all differentially expressed miRNAs and all upregulated miRNAs using TAM 2.0.

Target network and enrichment analysis was confined to validated 3′ UTR targets. The list of differentially expressed miRNAs was used as input to miRWalk v.3 (http://mirwalk.umm.uni-heidelberg.de/)^55^ using an interaction probability score of 0.95 (miRTarBase) to obtain the miRNA-mRNA target network. Cytoscape was then used to visualize networks and perform enrichment analysis.^55^ GO enrichment analysis was performed using the Cytoscape plugin BINGO (overrepresentation, hypergeometric test with Benjamini-Hochberg FDR correction, p = 0.05).

### EV proteomic analysis

EV isolates containing 25 μg protein were lysed using a solubilization buffer consisting of 8 M urea, 100 mM 4-(2-hydroxyethyl)-1-piperazineethanesulfonic acid (HEPES), 5% glycerol, and 0.5% n-dodecyl β-d-maltoside (DDS). Samples were reduced using Tris(2-carboxyethyl) phosphine (1.6 mM) and then alkylated with iodoacetamide (8 mM) for 55 min at room temperature. Proteins were digested using 0.45 μg trypsin/Lys-C solution (Promega) at room temperature for 20 h. 2 μL formic acid was then added to the samples, which were then desalted using C18 TopTips (Glygen) columns and finally vacuum dried. 5 μg protein were then analyzed by Orbitrap Fusion MS (Thermo Fisher Scientific).^56^ Peptides were separated by an in-house packed column (Polymicro Technology) using a water/acetonitrile/0.1% formic acid gradient. Samples were loaded onto the column for 105 min at a flow rate of 0.30 μL/min. Peptides were separated using successive rounds of acetonitrile at concentrations 2%–90% in a stepwise manner every 10 min. Peptides were eluted and sprayed into a mass spectrometer using positive electrospray ionization at an ion source. Peptide MS spectra (*m*/*z* 350–2,000) were acquired at a resolution of 60,000. Precursor ions were filtered according to monoisotopic precursor selection, and dynamic exclusion (30 s ± 10 ppm window). Fragmentation was performed with collision-induced dissociation in the linear ion trap. Precursors were isolated using a 2 *m*/*z* isolation window and fragmented with a normalized collision energy of 35%.

Differential protein expression analysis was performed using Perseus (https://maxquant.net/). Label-free quantitation values were log2 transformed for visual inspection using histogram distribution plots for each sample. Proteins identified in at least 2 of the 3 replicates were considered for analysis. A two-tailed Student’s t test with permutation-based FDR was used to calculate statistical significance between the two EV types. The log2 fold difference, p values, and log2 label-free quantitation values were used to make volcano plots and heatmaps using GraphPad Prism v.9.1 and RStudio (pheatmap package), respectively.

Functional annotations and pathway enrichment analysis of the whole proteome and differentially expressed proteins was performed using the Database for Annotation, Visualization, and Integrated Discovery (DAVID; v.6.8, https://david.ncifcrf.gov/) with *Homo sapiens* proteome as a background.^57^^,^^58^ Fisher’s exact test with multiple testing by the Benjamini-Hochberg method with an adjusted p value of 0.05 was used to extract significantly enriched terms. The top 10 functional GO terms of biological process, cellular component, molecular function, and KEGG pathways were extracted, with enrichment analysis performed on all proteins and upregulated proteins separately.

Protein-protein interaction analysis of differentially expressed proteins was performed using Search Tool for the Retrieval of Interacting Genes (STRING; v.11.5, https://string-db.org/cgi/input.pl) with a medium confidence score of 0.4.^59^ Cytoscape was then used to perform cluster and enrichment analysis. The Cytoscape plugin MCODE was used to perform cluster and enrichment analysis (degree cutoff = 2, cluster finding = haircut, node score cutoff = 0.2, K-core = 2, maximum depth = 100), while the cytoHubb plugin was used to identify to hub genes.

### Statistical analysis

All statistical tests and graphical depictions of data are defined within the respective materials and methods sections. Unless otherwise stated, all data are presented as mean ± standard error of the mean. To determine if differences existed in EV size and concentration between cell types, the data were analyzed by a one-way analysis of variance (ANOVA; GraphPad Prism v.9.1) with post hoc testing using Tukey’s multiple comparisons test. A final value of p ≤0.05 was considered significant for all analyses.

## Data availability

Data are available upon reasonable request.

## Supplemental information

Data S1. Tables S3, S4, and S6–S10

Document S2. Article plus supplemental information
