# Supplementary material for: Extracellular vesicle microRNA and protein cargo profiling in three clinical-grade stem cell products reveals key functional pathways
Source: Mol Ther Nucleic Acids. 2023 Mar 9;32:80–93. doi: 10.1016/j.omtn.2023.03.001 (PMC10034570; doi:10.1016/j.omtn.2023.03.001)
Supplement: Document S1. Figures S1–S6 and Tables S1, S2, S5, S11, and S12 [file mmc1.pdf]

OMTN, Volume *32*

Supplemental information

Extracellular vesicle microRNA and protein cargo profiling in three clinical-grade stem cell products reveals key functional pathways

Ramana Vaka, Sandrine Parent, Yousef Risha, Saad Khan, David Courtman, Duncan J. Stewart, and Darryl R. Davis

Table S1. Cell product characterization. The quality control parameters of cell products.

	HDC1	HDC2	HDC3	BM-MSC1	BM-MSC2	BM-MSC3	UC-MSC1	UC-MSC2	UC-MSC3
Population doublings in p1	5	5.5	6	6.1	6.3	6.2	3.8	3.9	4
Population doubling time (h)	33.7	30.7	28.2	31.4	22.7	27	25	30.6	22.6
% Viability post-thaw	95.7	94.2	95.6	94.2	94.6	89.5	94.1	94.3	95.3
Endotoxin Levels (EU/mL)	0.305	0.245	0.222	< 0.200	< 0.200	< 0.200	< 0.100	< 0.100	< 0.100
BACTEC Sterility (Aerobic and Anaerobic Bacterial growth)	No Growth	No Growth	No Growth	No Growth	No Growth	No Growth	No Growth	No Growth	No Growth
Mycoplasma	Negative	Negative	Negative	Negative	Negative	Negative	Negative	Negative	Negative

Table S2. List of differentially expressed miRNAs in HDC vs. BM-MSC EVs. Differential expression analysis of miRNAs between cell types was performed using ROSALIND® t-test method. A list of 56 differentially expressed (20 down regulated & 36 up regulated) miRNAs between HDC vs. BM-MSC EVs are shown in the table.

miRNA name	Description	Log2 Fold Change	p-Value
hsa-miR-1224-5p	MI0003764	3.51187	0.026825
hsa-miR-196a-5p	MI0000279	2.53976	0.005498
hsa-miR-630	MI0003644	2.30881	0.005956
hsa-miR-3140-5p	MI0014163	2.14175	0.000502
hsa-miR-874-5p	MI0005532	2.11602	0.049925
hsa-miR-1306-5p	MI0006443	2.07554	0.015506
hsa-miR-3131	MI0014151	2.04051	0.035703
hsa-miR-485-5p	MI0002469	2.00733	0.00846
hsa-miR-876-3p	MI0005542	1.94171	0.023983
hsa-miR-3196	MI0014241	1.84552	0.046151
hsa-miR-873-3p	MI0005564	1.81709	0.040183
hsa-miR-4741	MI0017379	1.77139	0.006722
hsa-miR-219a-2-3p	MI0000740	1.73822	0.044009
hsa-miR-34a-5p	MI0000268	1.73227	0.023769
hsa-miR-4787-3p	MI0017434	1.70069	0.028831
hsa-miR-520d-3p	MI0003164	1.674	0.005441
hsa-miR-501-3p	MI0003185	1.65044	0.04604
hsa-miR-590-3p	MI0003602	1.64296	0.047512
hsa-miR-10b-5p	MI0000267	1.53635	0.025041
hsa-miR-578	MI0003585	1.43806	0.038703
hsa-miR-519e-3p	MI0003145	1.43618	0.046268
hsa-miR-5196-5p	MI0018175	1.41843	0.030021
hsa-miR-199a-5p	MI0000281	1.41083	0.0096
hsa-miR-3690	MI0016091	1.37058	0.026933
hsa-miR-517a-3p	MI0003161	1.3701	0.035065
hsa-miR-619-3p	MI0003633	1.35175	0.034291
hsa-miR-568	MI0003574	1.27142	0.041672
hsa-miR-625-5p	MI0003639	1.07369	0.031
hsa-miR-4451	MI0016797	1.04325	0.037141
hsa-miR-1180-3p	MI0006273	1.04234	0.03193
hsa-miR-5010-3p	MI0017878	0.997418	0.022214
hsa-miR-302e	MI0006417	0.979929	0.046686
hsa-miR-1258	MI0006392	0.971695	0.026819
hsa-miR-197-5p	MI0000239	0.967794	0.035038
hsa-miR-92a-1-5p	MI0000093	0.900024	0.029612

hsa-miR-10a-5p	MI0000266	0.722411	0.031681
hsa-miR-16-5p	MI0000070	-0.634	0.003992
hsa-let-7g-5p	MI0000433	-0.646361	0.03895
hsa-miR-4286	MI0015894	-0.707782	0.008563
hsa-miR-199b-5p	MI0000282	-0.823786	0.025643
hsa-miR-19b-3p	MI0000074	-0.86821	0.043358
hsa-miR-24-3p	MI0000081	-0.893848	0.002479
hsa-miR-23b-3p	MI0000439	-0.999376	0.001002
hsa-miR-21-5p	MI0000077	-1.03304	0.035481
hsa-miR-376a-3p	MI0003529	-1.06254	0.042989
hsa-miR-337-5p	MI0000806	-1.19023	0.011657
hsa-miR-23a-3p	MI0000079	-1.2838	0.023105
hsa-miR-27b-3p	MI0000440	-1.31904	0.005334
hsa-let-7i-5p	MI0000434	-1.37666	0.002865
hsa-miR-4516	MI0016882	-1.41662	0.007115
hsa-miR-374a-5p	MI0000782	-1.44434	0.006919
hsa-miR-98-5p	MI0000100	-1.46162	0.013817
hsa-miR-22-3p	MI0000078	-1.59936	0.011553
hsa-miR-29a-3p	MI0000087	-1.63008	0.02321
hsa-miR-4454+hsa-miR-7975	MI0016800+ MI0025751	-1.63058	0.005845
hsa-miR-29b-3p	MI0000107	-3.46125	0.036546

Table S3. List of differentially expressed miRNAs in BM-MSC vs. UC-MSC EVs

Supplied as an Excel file.

Table S4. List of differentially expressed miRNAs in HDC vs. UC-MSC EVs

Supplied as an Excel file.

Table S5. List of differentially expressed proteins in HDC vs. BM-MSC EVs. Differential expression analysis of proteins between cell types was performed using Perseus (https://maxquant.net/perseus/). A list of 23 differentially expressed (9 down regulated & 14 up regulated) proteins between HDC vs. BM-MSC EVs are shown in the table.

Protein name	Gene name	Log2 Difference	p-Value
Ectonucleotide pyrophosphatase/phosphodiesterase family member 1;Alkaline phosphodiesterase I;Nucleotide pyrophosphatase	ENPP1	5.113114039	0.001777813
Caldesmon	CALD1	3.873648961	0.00057762
Neprilysin	MME	3.229970932	0.000944198
UTP--glucose-1-phosphate uridylyltransferase	UGP2	3.02215004	0.001529127
Major vault protein	MVP	2.45493571	0.000536644
Gamma-enolase;Enolase	ENO2	2.359129588	0.000297653
Niemann-Pick C1 protein	NPC1	2.298453013	0.002822243
Myosin regulatory light chain 12B;Myosin regulatory light chain 12A	MYL12B;MYL12A	2.242603302	0.000882352
Coronin-1C;Coronin	CORO1C	1.745135625	0.001159066
T-complex protein 1 subunit alpha	TCP1	1.660648346	0.002936757
Tubulin-specific chaperone A	TBCA	1.310288747	0.001436331
Protein spinster homolog 1	SPNS1	1.075093587	0.001325142
14-3-3 protein epsilon	YWHAE	0.795204798	0.000971929
Syntaxin-4	STX4	0.590283712	0.000162322
Band 4.1-like protein 2	EPB41L2	-2.013690313	0.000239126
Latent-transforming growth factor beta-binding protein 2	LTBP2	-2.616281509	0.002945221
Procollagen-lysine,2-oxoglutarate 5-dioxygenase 1	PLOD1	-3.127297719	0.002214353
Hemoglobin subunit beta;LVV-hemorphin-7;Spinorphin	HBB	-3.162877401	0.002488562
Fibrillin-1	FBN1	-3.394845327	9.5844E-06
Thrombospondin-2	THBS2	-3.841739019	0.002997381
Fibulin-1	FBLN1	-4.651089986	0.001453623
Versican core protein	VCAN	-5.202549616	0.000440219
Leucine-rich alpha-2-glycoprotein	LRG1	-5.707799911	0.000438009

Table S6. List of differentially expressed proteins in BM-MSC vs. UC-MSC EVs

Supplied as an Excel file.

Table S7. List of differentially expressed proteins in HDC vs. UC-MSC EVs

Supplied as an Excel file.

Table S8. List of differentially expressed miRNA-mRNA targets in HDC vs. BM-MSC EVs

Supplied as an Excel file.

Table S9. List of differentially expressed miRNA-mRNA targets in BM-MSC vs. UC-MSC EVs

Supplied as an Excel file.

Table S10. List of differentially expressed miRNA-mRNA targets in HDC vs. UC-MSC EVs

Supplied as an Excel file.

Table S11. Number of significantly enrichment terms of differentially protein cargo in BM-MSC or HDC or UC-MSC EVs. Functional enrichment analysis of the whole proteome and differentially expressed proteins was performed using DAVID v6.8 (https://david.ncifcrf.gov/). The total number of significantly enriched gene ontology terms of biological process (BP), cellular component (CC), and molecular function (MF) of the differentially expressed proteins between cell types are listed in the table.

**HDC vs. BM-MSC EV**	
Category	# of enriched Go terms
Biological process (BP)	6
Cellular component (CC)	15
Molecular function (MF)	7
**BM-MSC vs. UC-MSC EV**	
Category	# of enriched Go terms
Biological process (BP)	251
Cellular component (CC)	140
Molecular function (MF)	86
**HDC vs. UC-MSC EV**	
Category	# of enriched Go terms
Biological process (BP)	88
Cellular component (CC)	120
Molecular function (MF)	71

Table S12. Top 10 BP GO enrichment terms of HDC or BM-MSC or UC-MSC EV protein cargo. Functional enrichment analysis of the whole proteome and differentially expressed proteins was performed using DAVID v6.8 (https://david.ncifcrf.gov/). The top 10 significantly enriched biological process gene ontology terms from each cell product are listed in the table.

**HDC**			
GO term	Count	p-Value	Fold Enrichment
GO:0007155~cell adhesion	108	1.45E-36	4.080501516
GO:0002181~cytoplasmic translation	40	4.75E-28	9.35324833
GO:0050821~protein stabilization	47	8.64E-18	4.495936413
GO:0006412~translation	43	1.34E-14	4.057967605
GO:0070527~platelet aggregation	20	4.47E-14	9.35324833
GO:0016192~vesicle-mediated transport	41	1.33E-12	3.70316377
GO:0030335~positive regulation of cell migration	43	1.73E-12	3.534870219
GO:0036258~multivesicular body assembly	16	1.84E-12	10.86183677
GO:0007160~cell-matrix adhesion	27	3.62E-12	5.3103723
GO:0072659~protein localization to plasma membrane	33	4.27E-12	4.313532227
**BM-MSC**			
GO term	Count	p-Value	Fold Enrichment
GO:0002181~cytoplasmic translation	54	5.96E-47	12.68232711
GO:0006412~translation	58	1.77E-26	5.497570796
GO:0007155~cell adhesion	82	7.10E-20	3.111761889

GO:0050821~protein stabilization	48	1.28E-18	4.611755314
GO:0016192~vesicle-mediated transport	48	1.44E-17	4.354447077
GO:0006886~intracellular protein transport	51	2.06E-13	3.286578673
GO:0006457~protein folding	35	3.25E-13	4.456641054
GO:0070527~platelet aggregation	19	6.01E-13	8.924600561
GO:0015031~protein transport	57	1.29E-11	2.744467143
GO:1900026~positive regulation of substrate adhesion-dependent cell spreading	17	6.51E-11	8.166650035
**UC-MSC**			
GO term	Count	p-Value	Fold Enrichment
GO:0007596~blood coagulation	36	8.01E-27	10.61652
GO:0006958~complement activation, classical pathway	39	3.28E-24	8.16216
GO:0010951~negative regulation of endopeptidase activity	41	2.22E-23	7.287745
GO:0070527~platelet aggregation	25	1.31E-22	14.41749
GO:0007155~cell adhesion	71	1.25E-18	3.307999
GO:0006956~complement activation	17	2.86E-17	17.64701
GO:0007160~cell-matrix adhesion	29	3.35E-16	7.033579
GO:0030036~actin cytoskeleton organization	37	4.34E-16	5.275851
GO:0007229~integrin-mediated signaling pathway	28	7.12E-15	6.605832
GO:0042730~fibrinolysis	14	8.00E-15	19.12214

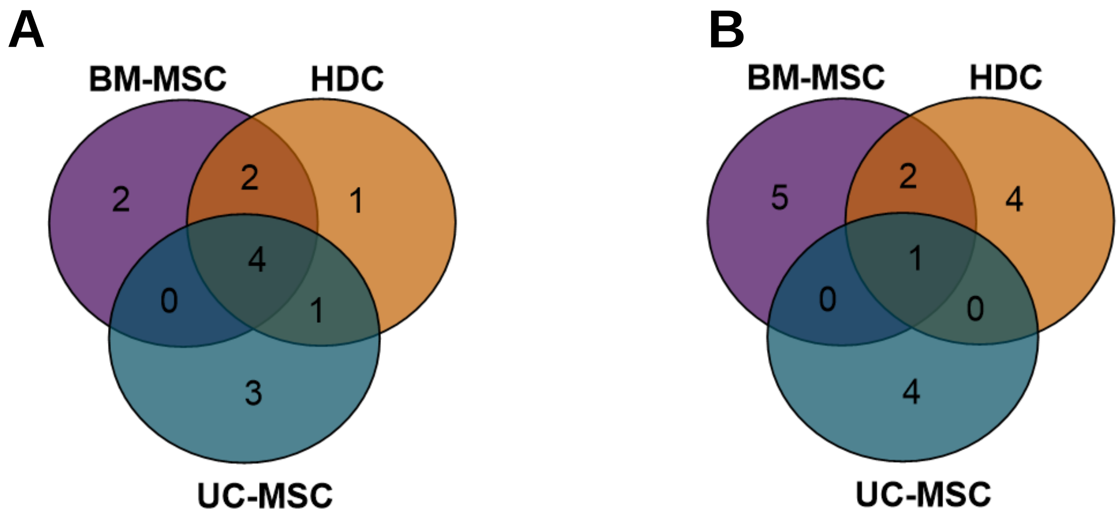

Figure S1. The highly abundant microRNAs and proteins in EVs.

(A) Venn diagram showing highly abundant (99th percentile) miRNAs. (B) Venn diagram showing highly abundant (99th percentile) proteins. BM-MSC: Bone marrow derived mesenchymal stromal cells, HDC: Heart derived cells, UC-MSC: Umbilical cord derived mesenchymal stromal cells.

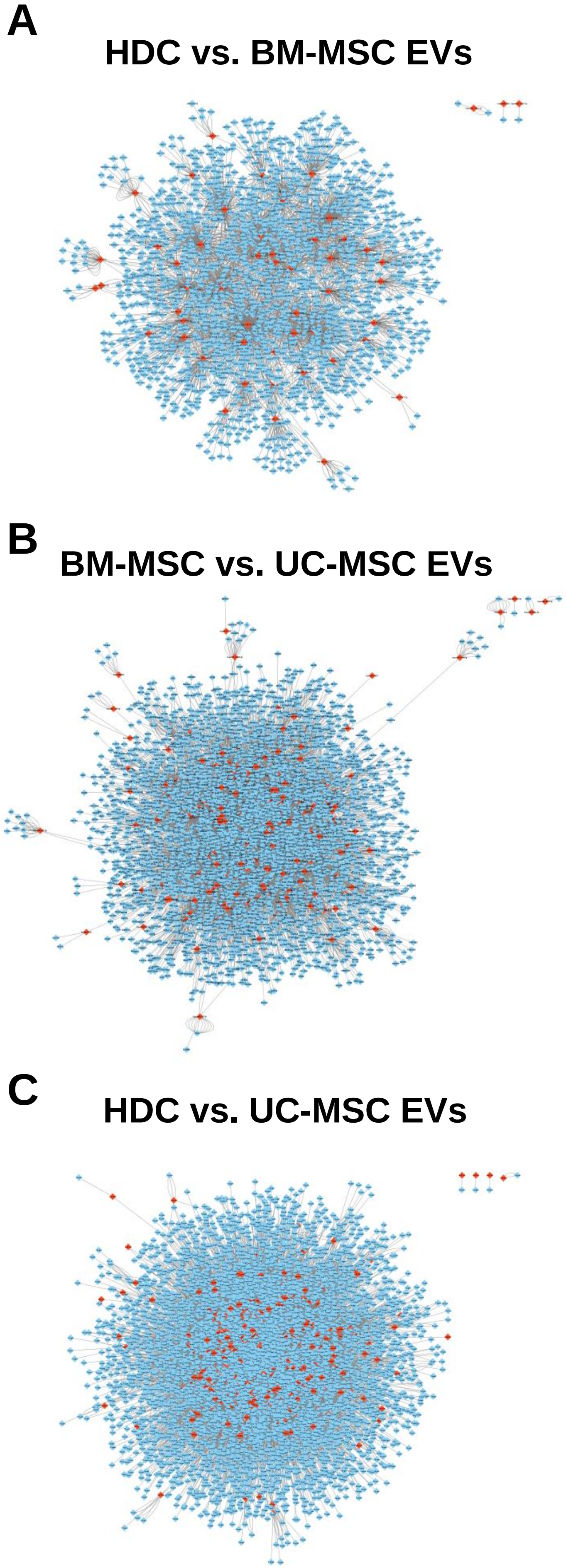
A
HDC vs. BM-MSC EVs
B
BM-MSC vs. UC-MSC EVs
C
HDC vs. UC-MSC EVs

Figure S2. MicroRNA-mRNA target regulatory networks.

Experimentally validated mRNA targets of differentially expressed miRNAs was obtained from miRWalk v3 (http://mirwalk.umm.uni-heidelberg.de/) (interaction probability score=0.95, miRTarBase, 3' UTR) and visualized in Cytoscape v3.9.1. (A) miRNA-mRNA target regulatory network of differentially expressed miRNAs from HDC vs. BM-MSC EVs. (B) miRNA-mRNA target regulatory network of differentially expressed miRNAs from BM-MSc vs. UC-MSC EVs. (C) miRNA-mRNA target regulatory network of differentially expressed miRNAs from HDC vs. UC-MSC EVs. Orange diamonds indicate miRNAs, light blue diamonds indicate mRNA targets. BM-MSC: Bone marrow derived mesenchymal stromal cells, HDC: Heart derived cells, UC-MSC: Umbilical cord derived mesenchymal stromal cells.

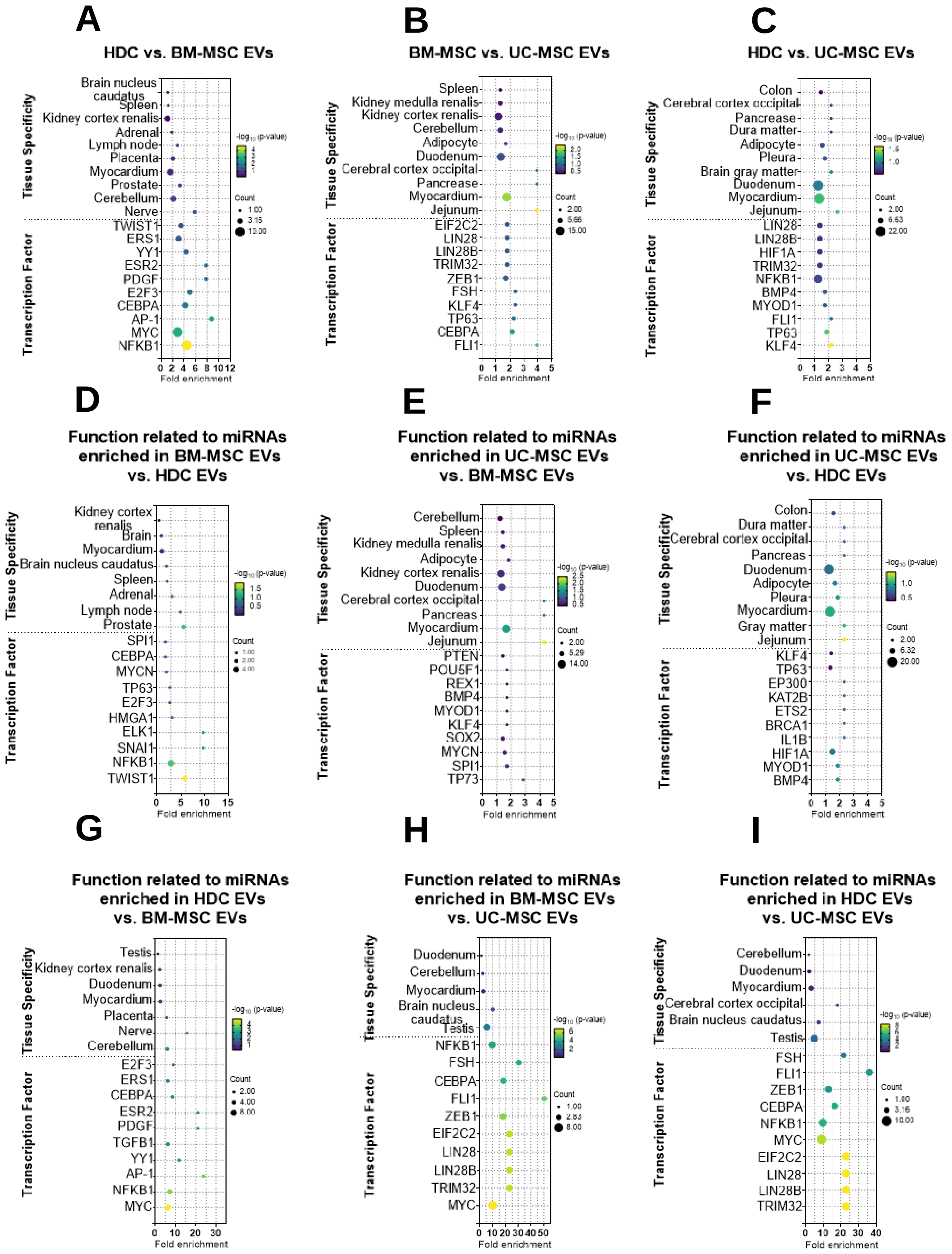
A
HDC vs. BM-MSC EVs
Tissue Specificity
Brain nucleus caudatus
Spleen
Kidney cortex renalis
Adrenal
Lymph node
Placenta
Myocardium
Prostate
Cerebellum
Nerve
Transcription Factor
TWIST1
ERS1
YY1
ESR2
PDGF
E2F3
CEBPA
AP-1
MYC
NFKB1
-log10 (p-value)
Count
1.00
3.16
10.00
Fold enrichment
B
BM-MSC vs. UC-MSC EVs
Tissue Specificity
Spleen
Kidney medulla renalis
Kidney cortex renalis
Cerebellum
Adipocyte
Duodenum
Cerebral cortex occipital
Pancrease
Myocardium
Jejunum
Transcription Factor
EIF2C2
LIN28
LIN28B
TRIM32
ZEB1
FSH
KLF4
TP63
CEBPA
FLI1
-log10 (p-value)
Count
2.00
5.66
16.00
Fold enrichment
C
HDC vs. UC-MSC EVs
Tissue Specificity
Colon
Cerebral cortex occipital
Pancrease
Dura matter
Adipocyte
Pleura
Brain gray matter
Duodenum
Myocardium
Jejunum
Transcription Factor
LIN28
LIN28B
HIF1A
TRIM32
NFKB1
BMP4
MYOD1
FLI1
TP63
KLF4
-log10 (p-value)
Count
2.00
6.63
22.00
Fold enrichment
D
Function related to miRNAs enriched in BM-MSC EVs vs. HDC EVs
Tissue Specificity
Kidney cortex renalis
Brain
Myocardium
Brain nucleus caudatus
Spleen
Adrenal
Lymph node
Prostate
Transcription Factor
SPI1
CEBPA
MYCN
TP63
E2F3
HMGA1
ELK1
SNAI1
NFKB1
TWIST1
-log10 (p-value)
Count
1.00
2.00
4.00
Fold enrichment
E
Function related to miRNAs enriched in UC-MSC EVs vs. BM-MSC EVs
Tissue Specificity
Cerebellum
Spleen
Kidney medulla renalis
Adipocyte
Kidney cortex renalis
Duodenum
Cerebral cortex occipital
Pancreas
Myocardium
Jejunum
Transcription Factor
PTEN
POU5F1
REX1
BMP4
MYOD1
KLF4
SOX2
MYCN
SPI1
TP73
-log10 (p-value)
Count
2.00
5.29
14.00
Fold enrichment
F
Function related to miRNAs enriched in UC-MSC EVs vs. HDC EVs
Tissue Specificity
Colon
Dura matter
Cerebral cortex occipital
Pancreas
Duodenum
Adipocyte
Pleura
Myocardium
Gray matter
Jejunum
Transcription Factor
KLF4
TP63
EP300
KAT2B
ETS2
BRCA1
IL1B
HIF1A
MYOD1
BMP4
-log10 (p-value)
Count
2.00
6.32
20.00
Fold enrichment
G
Function related to miRNAs enriched in HDC EVs vs. BM-MSC EVs
Tissue Specificity
Testis
Kidney cortex renalis
Duodenum
Myocardium
Placenta
Nerve
Cerebellum
Transcription Factor
E2F3
ERS1
CEBPA
ESR2
PDGF
TGFB1
YY1
AP-1
NFKB1
MYC
-log10 (p-value)
Count
2.00
4.00
8.00
Fold enrichment
H
Function related to miRNAs enriched in BM-MSC EVs vs. UC-MSC EVs
Tissue Specificity
Duodenum
Cerebellum
Myocardium
Brain nucleus caudatus
Testis
Transcription Factor
NFKB1
FSH
CEBPA
FLI1
ZEB1
EIF2C2
LIN28
LIN28B
TRIM32
MYC
-log10 (p-value)
Count
1.00
2.83
8.00
Fold enrichment
I
Function related to miRNAs enriched in HDC EVs vs. UC-MSC EVs
Tissue Specificity
Cerebellum
Duodenum
Myocardium
Cerebral cortex occipital
Brain nucleus caudatus
Testis
Transcription Factor
FSH
FLI1
ZEB1
CEBPA
NFKB1
MYC
EIF2C2
LIN28
LIN28B
TRIM32
-log10 (p-value)
Count
1.00
3.16
10.00
Fold enrichment

Figure S3. Functional enrichment analysis of EV miRNA cargo.

Functional enrichment analysis on all differentially expressed miRNAs or up- or down- regulated miRNAs between cell types was performed using TAM 2.0 (http://www.lirmed.com/tam2/) (overrepresentation, p-value ≤ 0.05). The TAM 2.0 data output on transcription factor & tissue specificity: enriched term, fold enrichment, p-values and miRNA count are graphed as bubble plots using GraphPad Prism v. 9.1. (A-C) The top 10 significantly enriched transcription factor & tissue specificity of all differentially expressed miRNAs between cell types. (D-I) The top 10 significantly enriched transcription factor & tissue specificity of enriched miRNAs in one cell type in comparison to other cell type. n=3 biological replicates. In a case the analysis yielded less than 10 enriched terms, all the terms are shown the in graphs. BM-MSC: Bone marrow derived mesenchymal stromal cells, HDC: Heart derived cells, UC-MSC: Umbilical cord derived mesenchymal stromal cells.

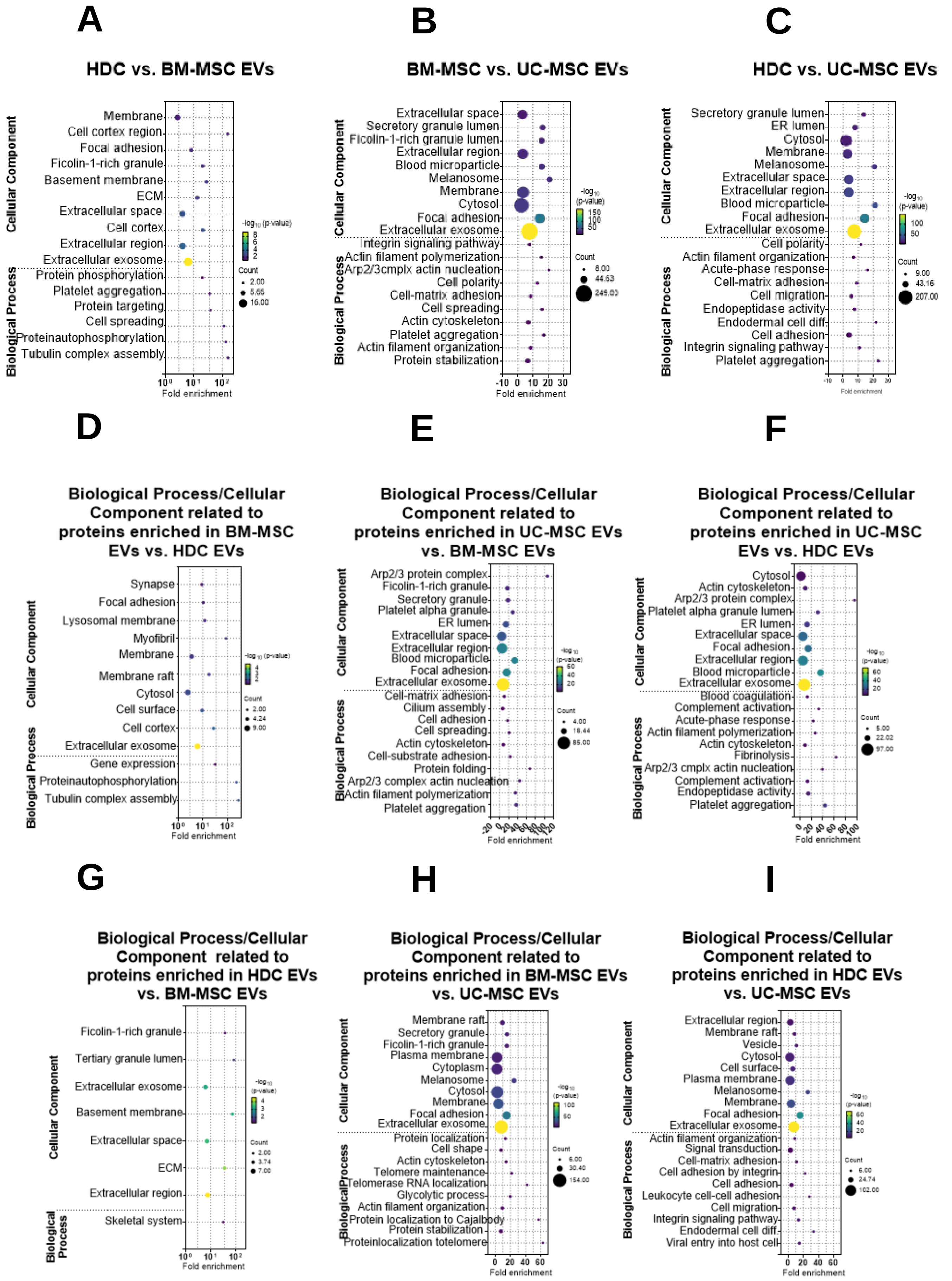
A
HDC vs. BM-MSC EVs
Cellular Component
Membrane
Cell cortex region
Focal adhesion
Ficolin-1-rich granule
Basement membrane
ECM
Extracellular space
Cell cortex
Extracellular region
Extracellular exosome
Biological Process
Protein phosphorylation
Platelet aggregation
Protein targeting
Cell spreading
Proteinautophosphorylation
Tubulin complex assembly
Fold enrichment
-log10 (p-value)
Count
B
BM-MSC vs. UC-MSC EVs
Cellular Component
Extracellular space
Secretory granule lumen
Ficolin-1-rich granule lumen
Extracellular region
Blood microparticle
Melanosome
Membrane
Cytosol
Focal adhesion
Extracellular exosome
Biological Process
Integrin signaling pathway
Actin filament polymerization
Arp2/3cmplx actin nucleation
Cell polarity
Cell-matrix adhesion
Cell spreading
Actin cytoskeleton
Platelet aggregation
Actin filament organization
Protein stabilization
Fold enrichment
C
HDC vs. UC-MSC EVs
Cellular Component
Secretory granule lumen
ER lumen
Cytosol
Membrane
Melanosome
Extracellular space
Extracellular region
Blood microparticle
Focal adhesion
Extracellular exosome
Biological Process
Cell polarity
Actin filament organization
Acute-phase response
Cell-matrix adhesion
Cell migration
Endopeptidase activity
Endodermal cell diff
Cell adhesion
Integrin signaling pathway
Platelet aggregation
Fold enrichment
D
Biological Process/Cellular Component related to proteins enriched in BM-MSC EVs vs. HDC EVs
Cellular Component
Synapse
Focal adhesion
Lysosomal membrane
Myofibril
Membrane
Membrane raft
Cytosol
Cell surface
Cell cortex
Extracellular exosome
Biological Process
Gene expression
Proteinautophosphorylation
Tubulin complex assembly
Fold enrichment
E
Biological Process/Cellular Component related to proteins enriched in UC-MSC EVs vs. BM-MSC EVs
Cellular Component
Arp2/3 protein complex
Ficolin-1-rich granule
Secretory granule
Platelet alpha granule
ER lumen
Extracellular space
Extracellular region
Blood microparticle
Focal adhesion
Extracellular exosome
Biological Process
Cell-matrix adhesion
Cilium assembly
Cell adhesion
Cell spreading
Actin cytoskeleton
Cell-substrate adhesion
Protein folding
Arp2/3 complex actin nucleation
Actin filament polymerization
Platelet aggregation
Fold enrichment
F
Biological Process/Cellular Component related to proteins enriched in UC-MSC EVs vs. HDC EVs
Cellular Component
Cytosol
Actin cytoskeleton
Arp2/3 protein complex
Platelet alpha granule lumen
ER lumen
Extracellular space
Focal adhesion
Extracellular region
Blood microparticle
Extracellular exosome
Biological Process
Blood coagulation
Complement activation
Acute-phase response
Actin filament polymerization
Actin cytoskeleton
Fibrinolysis
Arp2/3 cmplx actin nucleation
Complement activation
Endopeptidase activity
Platelet aggregation
Fold enrichment
G
Biological Process/Cellular Component related to proteins enriched in HDC EVs vs. BM-MSC EVs
Cellular Component
Ficolin-1-rich granule
Tertiary granule lumen
Extracellular exosome
Basement membrane
Extracellular space
ECM
Extracellular region
Biological Process
Skeletal system
Fold enrichment
H
Biological Process/Cellular Component related to proteins enriched in BM-MSC EVs vs. UC-MSC EVs
Cellular Component
Membrane raft
Secretory granule
Ficolin-1-rich granule
Plasma membrane
Cytoplasm
Melanosome
Cytosol
Membrane
Focal adhesion
Extracellular exosome
Biological Process
Protein localization
Cell shape
Actin cytoskeleton
Telomere maintenance
Telomerase RNA localization
Glycolytic process
Actin filament organization
Protein localization to Cajalbody
Protein stabilization
Proteinlocalization totelomere
Fold enrichment
I
Biological Process/Cellular Component related to proteins enriched in HDC EVs vs. UC-MSC EVs
Cellular Component
Extracellular region
Membrane raft
Vesicle
Cytosol
Cell surface
Plasma membrane
Melanosome
Membrane
Focal adhesion
Extracellular exosome
Biological Process
Actin filament organization
Signal transduction
Cell-matrix adhesion
Cell adhesion by integrin
Cell adhesion
Leukocyte cell-cell adhesion
Cell migration
Integrin signaling pathway
Endodermal cell diff
Viral entry into host cell
Fold enrichment

Figure S4. Functional enrichment analysis of EV protein cargo.

Functional enrichment analysis of the whole proteome and differentially expressed proteins was performed using DAVID v6.8 (https://david.ncifcrf.gov/) (H. sapiens proteome as a background, Fisher's exact test with multiple testing by the Benjamini-Hochberg method with adjusted p-value of 0.05). The significantly enriched functional Go terms of biological process (BP) and cellular component (CC) were extracted and graphed as bubble plots using GraphPad Prism v. 9.1. (A-C) The top 10 significantly enriched biological process (BP) and cellular component (CC) of all differentially expressed proteins among three cell products. (D-I) The top 10 significantly enriched biological process (BP) and cellular component (CC) of enriched proteins in one cell type in comparison to other cell type. n=3 biological replicates. BM-MSC: Bone marrow derived mesenchymal stromal cells, ER: Endoplasmic reticulum, diff: Differentiation, ECM: Extracellular matrix, HDC: Heart derived cells, UC-MSC: Umbilical cord derived mesenchymal stromal cells

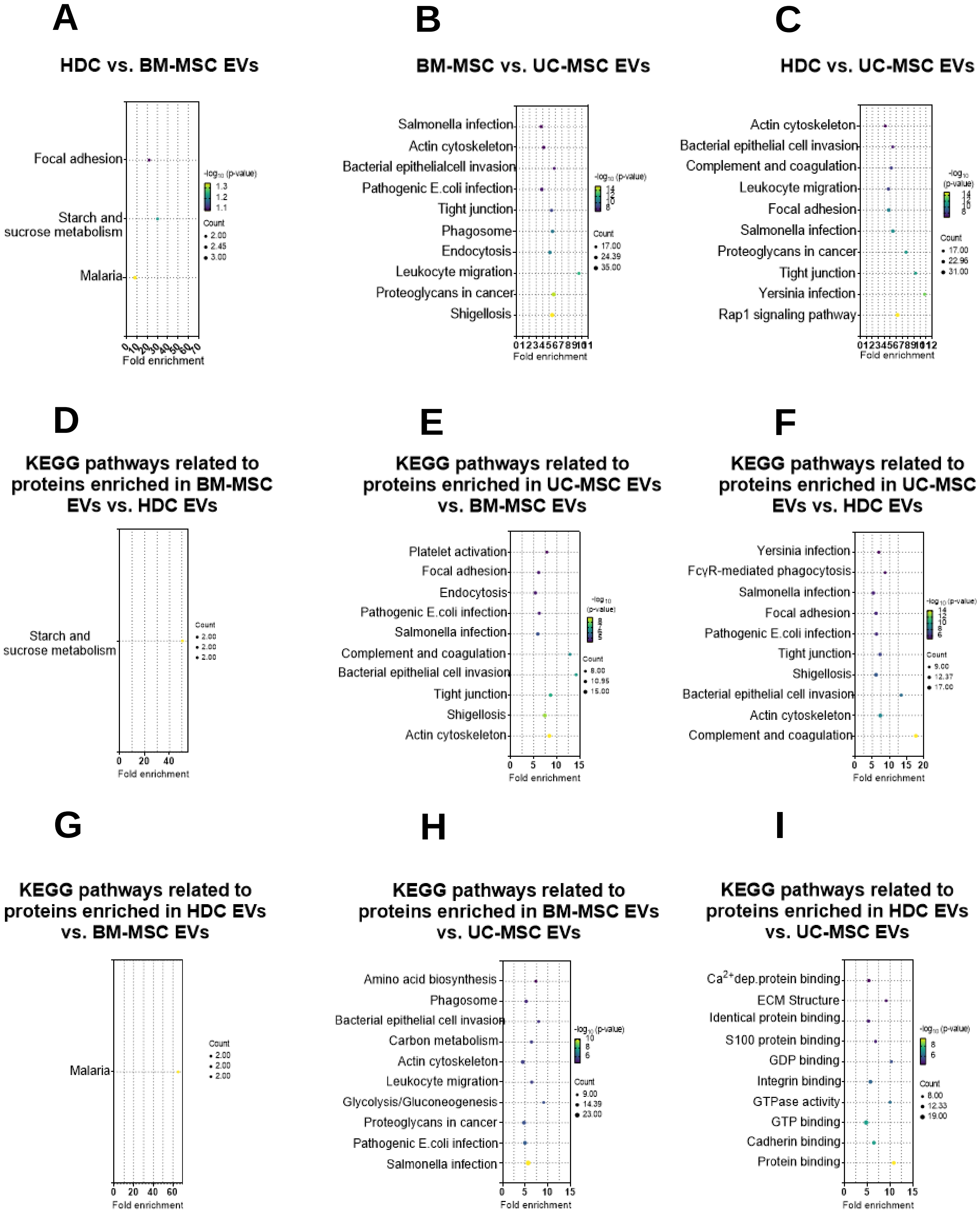
A
HDC vs. BM-MSC EVs
Focal adhesion
Starch and sucrose metabolism
Malaria
-log10 (p-value)
Count
Fold enrichment
B
BM-MSC vs. UC-MSC EVs
Salmonella infection
Actin cytoskeleton
Bacterial epithelialcell invasion
Pathogenic E.coli infection
Tight junction
Phagosome
Endocytosis
Leukocyte migration
Proteoglycans in cancer
Shigellosis
-log10 (p-value)
Count
17.00
24.39
35.00
Fold enrichment
C
HDC vs. UC-MSC EVs
Actin cytoskeleton
Bacterial epithelial cell invasion
Complement and coagulation
Leukocyte migration
Focal adhesion
Salmonella infection
Proteoglycans in cancer
Tight junction
Yersinia infection
Rap1 signaling pathway
-log10 (p-value)
Count
17.00
22.96
31.00
Fold enrichment
D
KEGG pathways related to proteins enriched in BM-MSC EVs vs. HDC EVs
Starch and sucrose metabolism
Count
2.00
2.00
2.00
0
20
40
Fold enrichment
E
KEGG pathways related to proteins enriched in UC-MSC EVs vs. BM-MSC EVs
Platelet activation
Focal adhesion
Endocytosis
Pathogenic E.coli infection
Salmonella infection
Complement and coagulation
Bacterial epithelial cell invasion
Tight junction
Shigellosis
Actin cytoskeleton
-log10 (p-value)
Count
8.00
10.95
15.00
0
5
10
15
Fold enrichment
F
KEGG pathways related to proteins enriched in UC-MSC EVs vs. HDC EVs
Yersinia infection
FcγR-mediated phagocytosis
Salmonella infection
Focal adhesion
Pathogenic E.coli infection
Tight junction
Shigellosis
Bacterial epithelial cell invasion
Actin cytoskeleton
Complement and coagulation
-log10 (p-value)
Count
9.00
12.37
17.00
0
5
10
15
20
Fold enrichment
G
KEGG pathways related to proteins enriched in HDC EVs vs. BM-MSC EVs
Malaria
Count
2.00
2.00
2.00
0
20
40
60
Fold enrichment
H
KEGG pathways related to proteins enriched in BM-MSC EVs vs. UC-MSC EVs
Amino acid biosynthesis
Phagosome
Bacterial epithelial cell invasion
Carbon metabolism
Actin cytoskeleton
Leukocyte migration
Glycolysis/Gluconeogenesis
Proteoglycans in cancer
Pathogenic E.coli infection
Salmonella infection
-log10 (p-value)
10
8
6
Count
9.00
14.39
23.00
0
5
10
15
Fold enrichment
I
KEGG pathways related to proteins enriched in HDC EVs vs. UC-MSC EVs
Ca2+dep.protein binding
ECM Structure
Identical protein binding
S100 protein binding
GDP binding
Integrin binding
GTPase activity
GTP binding
Cadherin binding
Protein binding
-log10 (p-value)
8
6
Count
8.00
12.33
19.00
0
5
10
15
Fold enrichment

Figure S5. KEGG enrichment analysis of EV protein cargo.

Functional enrichment analysis on all differentially expressed proteins or up- or down- regulated proteins between cell types was performed using DAVID v6.8 (https://david.ncifcrf.gov/) (H. sapiens proteome as a background, Fisher's exact test with multiple testing by the Benjamini-Hochberg method with adjusted p-value of 0.05). DAVID Kyoto Encyclopedia of Genes and Genomes (KEGG) pathway output: enriched term, fold enrichment, p-values and protein count are graphed as bubble plots using GraphPad Prism v. 9.1. (A-C) The top 10 significantly enriched KEGG pathways of all differentially expressed proteins among three cell products. (D-I) The top 10 significantly KEGG pathways of enriched proteins in one cell type in comparison to other cell type. n=3 biological replicates. In a case the analysis yielded less than 10 enriched terms, all the terms are shown the in graphs. BM-MSC: Bone marrow derived mesenchymal stromal cells, E.Coli: Escherichia Coli, ECM: Extracellular matrix, GDP: Guanosine diphosphate, GTP: Guanosine triphosphate, HDC: Heart derived cells, UC-MSC: Umbilical cord derived mesenchymal stromal cells.

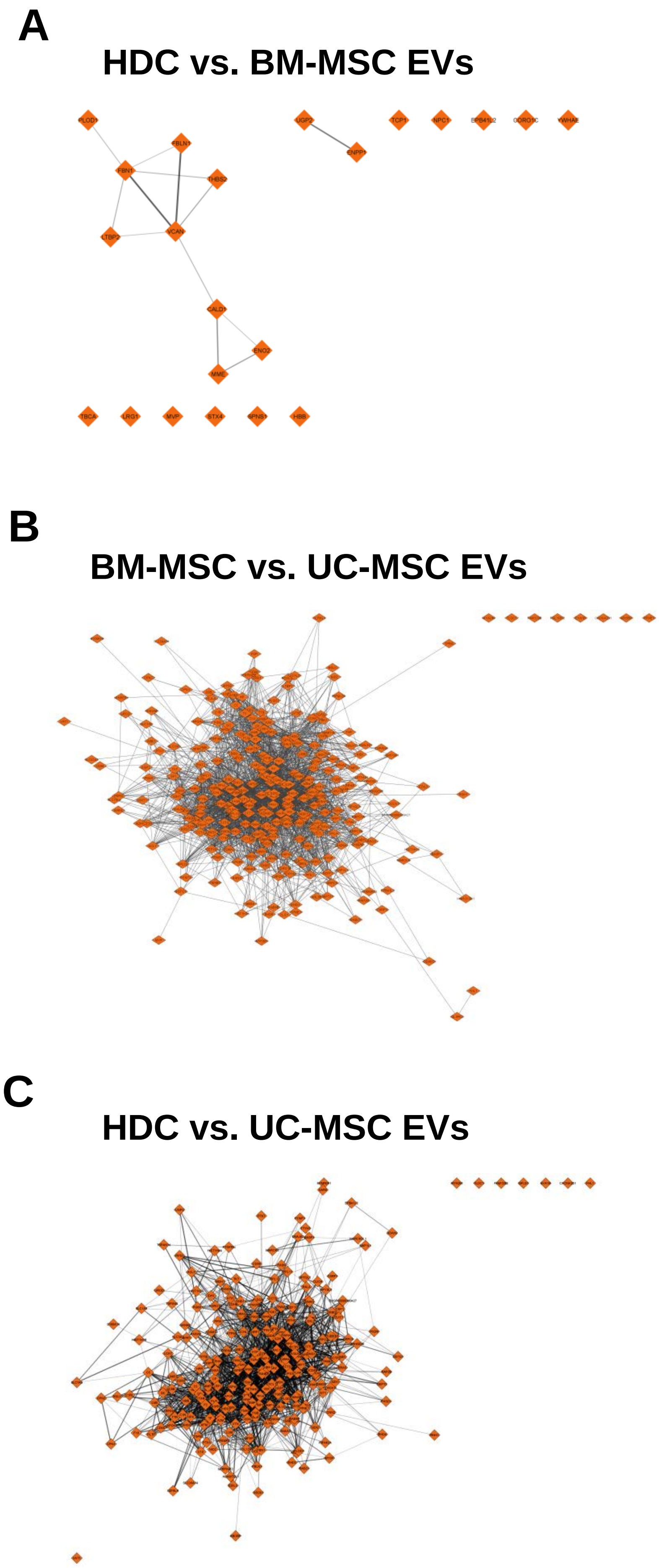
A
HDC vs. BM-MSC EVs
PLOD1
FBLN1
FBN1
THBS2
LTBP2
VCAN
CALD1
ENO2
MME
UGP2
ENPP1
TCP1
NPC1
EPB41L2
CORO1C
YWHAE
TBCA
LRG1
MVP
STX4
SPNS1
HBB
B
BM-MSC vs. UC-MSC EVs
C
HDC vs. UC-MSC EVs

Figure S6. Protein-protein interaction (PPI) networks.

PPI network analysis of differentially expressed proteins between cell types was performed by STRING v11.5 (https://string-db.org/cgi/input.pl) (medium confidence score=0.4). and Cytoscape v3.9.1. STRING PPI networks were visualized in Cytoscape. (A) PPI network of differentially expressed proteins in HDC vs. BM-MSC EVs. (B) PPI network of differentially expressed proteins in BM-MSC vs. UC-MSC EVs. (C) PPI network of differentially expressed proteins in HDC vs. UC-MSC EVs. The orange color diamonds indicate nodes, gray lines indicate edges.
